# COVID-19 IDD: Findings from a global survey exploring family members’ and paid staff’s perceptions of the impact of COVID-19 on individuals with intellectual and developmental disabilities (IDD) and their caregivers.

**DOI:** 10.12688/hrbopenres.13497.1

**Published:** 2022-04-04

**Authors:** Christine Linehan, Gail Birkbeck, Tal Araten-Bergman, Jennifer Baumbusch, Julie Beadle-Brown, Christine Bigby, Valerie Bradley, Michael Brown, Femmianne Bredewold, Masauso Chirwa, Jialiang Cui, Marta Godoy Gimenez, Tiziano Gomeiro, Šárka Kanova, Thilo Kroll, Henan Li, Mac MacLachlan, Jayanthi Narayan, Finiki Nearchou, Adam Nolan, Mary-Ann O'Donovan, Flavia H Santos, Jan Šiška, Tim Stainton, Magnus Tideman, Jan Tossebro

**Affiliations:** 1 UCD Centre for Disability Studies, University College Dublin, Belfield, Dublin 4, Ireland; 2 Business Information Systems, O'Rahilly Building, University College Cork, Cork, Ireland; 3 Living with Disability Research Centre, School of Allied Health, Human Services & Sport,, La Trobe University, Bundoora Vic 3086, Australia; 4 Canadian Institute for Inclusion and Citizenship, University of British Columbia, 2080 West Mall, Vancouver, BC Canada V6T 1Z2, Canada; 5 Tizard Centre, Univesity of Kent, Canterbury, Kent, CT2 7NZ, UK; 6 Human Services Research Institute, 2336 Massachusetts Ave,, Cambridge, MA, MA 02140, USA; 7 School of Nursing and Midwifery, Queen's University, Belfast, BT9 7BL, UK; 8 University of Humanistic Studies, Kromme Nieuwegracht 29, Utrecht, 3512 HD, The Netherlands; 9 School of Humanities and Social Sciences, Department of Social Work & Sociology, University of Zambia, Great East Road Campus, P.O.Box 32379, Lusaka, 10101, Zambia; 10 Department of Social Work, The Chinese University of Hong Kong, Shatin, New Territories, Hong Kong, China; 11 Department of Psychology, University of Almeria, La Canada de San Urbano, Almeria, 04120, Spain; 12 ANFFAS Trentino Onlus DAD© project group, Trento, Trentino, 38121, Italy; 13 Department of Education, University of West Bohemia, Plzeň 3, 301 00, Czech Republic; 14 UCD School of Nursing, Midwifery, and Health Systems, University College Dublin, Belfield, Dublin 4, Ireland; 15 School of Psychology, Maynooth University, Maynooth, Ireland; 16 Inclusive Education at Faculty of Health, Education and Society, University of Northampton, Northampton, UK; 17 UCD School of Psychology, University College Dublin, Belfield, Dublin, Ireland; 18 Centre for Disability Studies, Sydney Medical School, Faculty of Medicine and Health,, University of Sydney, Sydney, Camperdown NSW 2050, Australia; 19 Department of Special Education,, Charles University, Praha 1, 116 39, Czech Republic; 20 Department of Social Sciences, Ersta Sköndal Bräcke University, Sköndal, Sweden; 21 School of Health and Welfare, Halmstad University, Halmstad, Sweden; 22 Department of Social Work, Norwegian University of Science and Technology, Trondheim, Norway

**Keywords:** Caregivers, Carers, Coronavirus, COVID-19, Health Disparity, Intellectual and Developmental Disability, Intellectual Disability, Pandemic

## Abstract

**Background:** A growing body of evidence attests to the disproportionate impact of COVID-19 on persons with intellectual and developmental disabilities (IDD) during the pandemic. This study asked caregivers about their perceptions of how COVID-19 impacted them and the people they support.

**Method:** An online survey was conducted in 12 countries during August-September 2020 and sought information on demographics, support practices, information and training, experiences of COVID-19, social distancing, and wellbeing, as measured by the DASS12. This study reports on 3,754 family members, direct support professionals, and managers who participated in the survey.

**Results:** Caregivers observed increases in depression/anxiety, stereotyped behaviours, aggression towards others and weight gain in the person(s) they supported. They also reported difficulties supporting the person(s) to access healthcare.  Families reported reducing or ceasing employment and absorbed additional costs when supporting their family member. Direct support professionals experienced changes in staff shifts, staff absences, increased workload and hiring of casual staff. Caregivers’ wellbeing revealed high levels of stress, depression, and less so anxiety. The strongest predictor of wellbeing among families was observation of changes in mood in the person(s) they supported, while for direct support professionals, the strongest predictors of wellbeing were reorganisation of staff shifts and increases in new direct support staff.

**Discussion:** Findings support the contention of this population experiencing a disproportionate burden during the COVID-19 pandemic, reflecting historical inequities in access to healthcare and other human rights violations which are now protected under the United Nations Convention on the Rights of Persons with Disabilities.

## Introduction

### Background to this study

The United Nation’s Convention on the Rights of Persons with Disabilities affirms the right of persons with disabilities to full inclusion and participation in all aspects of life, charging signatories to the Convention with organising, strengthening, and extending support services ( United Nations, 2006). Quality of life outcomes for individuals with intellectual and developmental disabilities (IDD) are a function of the support they receive, with the American Association on Intellectual and Developmental Disabilities’ support needs model arguing *“*put another way, if supports were removed, people with ID (intellectual disability) would not be able to function as successfully in typical activities and settings” ( American Association for Intellectual and Developmental Disability, 2021). The COVID-19 pandemic has disrupted access to the supports typically received by people with IDD and has placed additional challenges on mainstream systems to make adjustments to accommodate their needs. This study reports on family members’ and paid staff’s perceptions of the impact of the COVID-19 pandemic on people with IDD and their caregivers.

Current evidence on the impact of the pandemic on persons with IDD and their caregivers can be broadly classified into three domains (1) diagnosis, risk factors and mortality for COVID-19 among people with IDD; (2) access to healthcare, vaccines, and potentially discriminatory practices; and (3) proxy and self-reported impact by persons with IDDs, family, and staff. A selection of emerging evidence from these studies is presented below. 

### Diagnosis, risk factors, and mortality

Persons with IDD report a higher incidence of COVID-19 (3.1% vs 0.9%), higher levels of hospitalisation for COVID-19 (63.1% vs 29.1%), and higher mortality (8.2% vs 3.8%) when compared with the general population ( Gleason *et al.,* 2021). Multiple factors contribute to the higher vulnerability of persons with IDD to COVID-19: pre-existing comorbid conditions ( Landes *et al*., 2020a); the shift-based nature of staff support ( Ervin, 2021); poor health literacy, difficulties coping with a lack of routine, and difficulties adhering to social distancing and mask wearing ( Buonaguro & Bertelli, 2021; Gleason *et al.,* 2021; Siasoco, 2020). Risk factors for admissions of persons with IDD to hospital with COVID-19 were identified as the presence of comorbid conditions and male gender ( Mills *et al.,* 2020) and IDD itself was identified as a risk factor for admission to ICU ( Temperoni *et al.,* 2021). 

Mortality rates, mostly from US studies, reveal that for those aged under 70 years, individuals with developmental disorders had the highest odds of death (odds ratio (OR)=3.1; 95% CI 1.5-6.0), and those with intellectual disability (ID) the third highest odds (2.75; 95% CI 1.6-4.6) ( Fair Health, 2020). ID was also reported as the strongest risk factor, apart from age, of COVID-19 mortality ( Gleason *et al.,* 2021). Mortality rates among the IDD population in New York recorded 1,175 per 100,000 which is markedly inflated from the 151 per 100,000 for the general New York population ( Landes *et al.,* 2020b). A study across eight states in the US which found similar COVID-19 rates among those with and without IDD, reported that while 6.7% of the general population who were diagnosed with COVID-19 subsequently died, this figure rose to 12.3% among those with IDD ( Spreat *et al.,* 2020).

People with IDD were reported to die from COVID-19 at a younger age than the general population ( Perera *et al.,* 2020; Turk *et al.,* 2020), and were reported as more likely to die from COVID-19 if they lived in congregated settings ( Landes *et al*., 2021). Despite congregated living being a known risk factor for COVID-19 ( Buono *et al.,* 2021; Courtenay & Perera, 2020) one global survey reported over two-thirds of people with disabilities were restricted or denied the right to leave these settings during the pandemic ( Brennan *et al.,* 2020), a finding that reflects concerns regarding human rights violations during the pandemic ( European Association of Service Providers for Persons with Disabilities, 2020; Hughes & Anderson, 2020). Despite these elevated risk factors, the absence of attention in public health measures towards people with disabilities was marked ( Sabatello *et al*., 2020).

### Access to healthcare, vaccines, and potentially discriminatory practices

There is evidence that access to healthcare during the pandemic was compromised for those with IDD ( Jeste *et al.,* 2020; Rosencrans *et al.,* 2021). Many disability and healthcare providers turned to virtual support, the advantages of which include convenience, and the possibility for healthcare professionals to become more familiar with individuals’ home environments ( Keller, 2021). Concerns have, however, been expressed regarding the effectiveness of virtual support if persons with IDD are not physically present at consultations ( Lunsky *et al.,* 2021a). Notwithstanding these challenges, some people with IDD have expressed a preference for virtual consultations post-pandemic ( Moriarta *et al*., 2020). Access to COVID-19 testing for persons with IDD was monitored in a review of COVID-19 health and social care policies in 15 European countries, many of whom failed to prioritise persons with IDD living in residential care ( Oakley *et al.,* 2020). Concerns were expressed regarding the challenges of testing people with IDD, and whether adequate support was provided to enable persons with IDD understand and communicate symptoms ( Sulkes, 2020), with some commentators calling for a ‘higher index of suspicion’ of symptoms being necessary for those with IDD ( Tenenbaum *et al.,* 2021). Triage protocols have been found to exclude people with IDD based on disability rather than risk, a practice which resulted in investigations following complaints being filed in numerous US states ( Department of Health and Human Services, 2021; Felt *et al.,* 2021). Concerns have also been expressed regarding the availability of accommodations for individuals with IDD who may be hospitalised for COVID-19, notably whether a caregiver was permitted to accompany the individual or was deemed a ‘visitor’ ( Margolis, 2021; MacGregor, 2021). Visitor status left some individuals with IDD unsupported during hospitalisation due to visitor restrictions and could be deemed a violation of the United Nations Convention on the Rights of Persons with Disabilities.

A small number of studies examined vaccine uptake, and notably whether persons with IDD faced discrimination if knowingly supported by vaccine-hesitant caregivers. US and Canadian studies reveal conflicting data regarding the intention of support workers to vaccination ( Iadarola *et al.,* 2021; Lunsky *et al.,* 2021c; Unroe *et al.,* 2021), although vaccine hesitancy findings have prompted national campaigns to promote reliable information on vaccination ( National Association of Councils on Developmental Disabilities, 2021). A large longitudinal study of direct support workers in the US identified 69% as being fully vaccinated, and a fear that vaccination was unsafe as the main reason for not being vaccinated ( Institute on Community Integration, 2021). An additional area of potential discrimination for those with IDD is the routine exclusion of individuals with cognitive impairment in randomised control trials (RCT) for COVID-19 treatments ( Barco *et al.,* 2020; Tornero *et al.,* 2020), a practice which fails to acknowledge the assumption of capacity principle driving assisted or supported decision-making legislation ( Murphy *et al.,* 2020).

### Self- and proxy-reported impact of COVID-19 on the mental health and social needs of people with IDD and their caregivers

A small number of studies have reported on the impact of the pandemic as directly experienced by individuals with IDD. Spanish data surveying 582 individuals with IDD during the pandemic revealed raised levels of anxiety, concerns regarding employment and one in five feeling unsupported ( Amor *et al.,* 2021), with those living in the family home reporting they relied heavily on their natural supports ( Navas *et al.,* 2021). A large US study of individuals with intellectual and cognitive disabilities found that one in four were unable to maintain their professional support throughout the pandemic, and access to regular healthcare was compromised for more than half, albeit access to prescriptions was rarely hindered. Almost half felt anxious or depressed, with one in five of these stating that they did not have sufficient access to emotional support ( Drum *et al.,* 2020). These findings are echoed by the US National Core Indicator survey of almost 3,000 family members supporting an adult with intellectual disability in their household who reported that almost half felt the support changes introduced by the pandemic were “not good for my family” ( National Core Indicators, 2021). Emotional distress was also reported by the Irish Intellectual Disability Supplement to the Irish Longitudinal Study on Ageing (IDS-TILDA) longitudinal study of older people with ID where half of all 692 participants with ID or their proxy respondents reported being stressed or anxious due to COVID-19 ( McCarron *et al.,* 2021).

The emotional and mental health impact of the pandemic described by persons with IDD is reflected in studies examining uptake of mental health supports for this population. A UK survey of ID mental health services observed increases in urgent psychiatric consultations to address a deterioration in mental health and behaviour ( Rauf *et al.,* 2021), similar to increases observed by a Dutch online support service ( Zaagsma *et al.,* 2020). Both observational and family-report studies reveal significant increases in challenging behaviour during lockdown ( Schuengel *et al.,* 2020; Wieting *et al.,* 2021) which in turn led to a negative appraisal of persons with IDD ( Murray *et al.,* 2021). While the disruption to support services for persons with IDD throughout the pandemic was wide ranging, it was deemed to have a specific detrimental impact for those who engage in behaviours that challenge ( Gleason *et al.,* 2021).

A number of studies examined the impact of the pandemic on family members of persons with IDD. Although a longitudinal study interrupted by the pandemic reported no impact on parental and child wellbeing ( Bailey *et al.,* 2021), other studies reported high levels of anxiety and depression among family caregivers ( Willner *et al.,* 2020). The National Core Indicator’s family survey revealed concerns regarding the diminution of supports and threats to wellbeing due to loss of employment and income ( National Core Indicators, 2021). Qualitative evidence from Dutch maternal interviews revealed mothers’ anxieties as to whether their children would survive COVID-19; these concerns were exacerbated by a belief that medical professionals would value their children less than those without disabilities ( Embregts *et al.,* 2021). Despite these fears, these mothers reported a ‘calmness’ to their family life which they attributed to a lessening of demands during the pandemic ( Embregts *et al.,* 2021). Other positives reported by parents and siblings included the ‘silver lining’ of spending more time with their family member ( Neece *et al*., 2020; Redquest *et al.,* 2021).

Disrupted access to mental health, physical health and social support services was a key concern of senior representatives in UK and Irish IDD services ( Tromans *et al.,* 2020). Challenges for direct support professionals, captured in a Dutch qualitative study, reveal an emotional toil, notably of anxiety from a fear of getting COVID-19, frustration at being asked to take what they perceived as inappropriate risks within the workplace, and concern regarding the responsibility they felt for those they were supporting ( Embregts *et al.,* 2020). These findings are echoed in a Canadian study reporting 34% of direct support professionals reached the screen threshold for anxiety, 21% for depression, and 25% for moderate to severe levels of clinical distress ( Lunsky *et al.,* 2021b). Similar findings emerged from an Irish study which revealed mild levels of anxiety and depression, and moderate levels of personal and work-related burnout, with staff supporting those in independent living arrangements being particularly at risk ( McMahon *et al.,* 2020). Staff concerns were found to vary by residential settings in a survey of UK staff which revealed that those supporting persons with ID in congregated settings prioritised infection as a cause for concern, while those supporting individuals in the community prioritised remote support ( Sheehan *et al.,* 2021).

### The present study

The present study sought to explore globally family members’ and paid staff’s perceptions of the impact of COVID-19 on the individuals with IDD they support and explore their own experiences as caregivers via an anonymous online survey conducted in 18 jurisdictions (Australia, Brazil, Canada, Czech Republic, Germany, Greece, Hong Kong SAR, India, Ireland, Israel, Italy, the Netherlands, Norway, Portugal, Sweden, United Kingdom (UK), United States (US), and Zambia). As outlined in the study protocol ( Linehan *et al.,* 2020), caregivers’ perception of outcomes for people with IDD was explored generally throughout the online survey, but specifically in questions relating to access to health services and protective equipment, continuity of care, adverse impact of restrictions, and questions relating to their experiences of symptoms, testing and treatment. The perception of outcomes for caregivers was also explored generally throughout the survey, and specifically in questions relating to mood and impact, using the Depression, Anxiety, and Stress Scale and the Coronavirus Anxiety Scale, and questions relating to their experiences of symptoms, testing, and treatment.

### Research questions

The study protocol identifies two core research questions. Firstly, what are family members' and paid staff’s perceptions of the impact of the COVID-19 pandemic on individuals with IDD and their caregivers? Secondly, do differences exist in the self-reported experiences of those supporting individuals living in different living arrangements and in different international jurisdictions? This paper reports descriptively and statistically on the impact of the pandemic as perceived by these caregivers. Statistical analyses were also undertaken to explore differences in perceived impact among those supporting individuals in different living arrangements. The proposal cited in the study protocol to examine differences in impact across different jurisdictions was discussed among co-investigators post data collection. Co-investigators, who were responsible for data collection within their own jurisdictions, agreed that such an analysis would be inappropriate given that the sample sizes for each nationality are not representative of their country and therefore not comparable. For this reason, this paper focuses on the global rather than comparative findings.

## Methods

As an online survey was used in this study, the Checklist for Reporting Results of Internet E-Surveys (CHERRIES) informs the presentation of methodology. Devised by Eysenbach (2004), these guidelines comprise eight categories (1) design (2) institutional review board approval and informed consent process (3) development and pre-testing of survey (4) recruitment process and description of the sample having access to the survey (5) survey administration (6) response rates (7) preventing multiple responses from the same individual and (8) data analysis.

### (1) Design

This study employed a cross-sectional design using an anonymous online survey of caregivers to gather data on their experiences and the experiences of the individuals with IDD they supported during the pandemic. A study protocol details the proposed methodology prior data collection ( Linehan *et al.,* 2020)

### (2) Institutional review board approval and informed consent process

Ethical approval was awarded by University College Dublin’s (UCD) Human Research Ethics Committee - Humanities (HREC-HS) as lead investigator of the study (application HS-20-28). Co-investigators, who led the launch of the survey in their own countries, were supplied with confirmation of UCD’s ethical approval and were requested to ensure that approval was secured for the study according to local practices. The study’s informed consent process was compliant with HREC’s guidance which requires all research participants to: firstly, be presented with an electronic Information Sheet providing details of the study; secondly, endorse a statement of consent before being directed to the survey; and thirdly, be provided with a list of national and/or local support services in the event any participant might become distressed during the completion of the survey.

To preserve the anonymity of participants in this study and adhering to the ethical approval awarded for this survey by University College Dublin, participants were asked to click an affirmative response to the statement below, which was presented at the beginning of the online survey, to indicate their written informed consent. The statement read; “please indicate your agreement with each of the statements below to proceed with the COVID-19 survey:

I am 18 years or older. 

I have read the Information Sheet for this study. 

I am satisfied that I received enough information on this study.

I know that I can quit at any time by exiting the survey.

I give my consent to take part in this survey.”

### (3) Development and pre-testing of survey

A bespoke survey was designed comprising eight sections gathering data on (1) demographics (2) management practices (3) direct support professional practices (4) family practices (5) information and training about COVID-19 (6) experience, if any, of COVID-19 for caregivers and the people with IDD they support (7) impact of social distancing (8) caregiver wellbeing.

In addition to self-developed items, the survey included two standardised and well-validated measures; the modified Depression, Anxiety, and Stress Scale (DASS12; Ang *et al.,* 2018; Osman *et al.,* 2014) and the Coronavirus Anxiety Scale (CAS; Lee, 2020). DASS12 comprises a 12-item scale which screens for depression, anxiety, and stress. Each item presents a statement such as ‘I found it very hard to wind down’ which is rated using a four-point rating scale from ‘0=did not apply to me at all’ through to ‘3=applied to me very much or most of the time.’ Following Ang *et al.* (2018) each participant’s total score for depression, anxiety, and stress was transformed into categories of normal (scoring zero), mild (scoring 1-2) moderate (scoring 3-4), or severe (scoring 5-12). CAS comprises five items, for example, ‘I had trouble falling asleep or staying asleep because I was thinking about the coronavirus.’ Respondents rate each item using a five-point rating scale ranging from ‘0=not at all’ to ‘4=nearly every day over the last two weeks.’ Higher scores indicate greater levels of anxiety.

The entire survey comprised 269 items, all of which were closed items employing either nominal category responses or rating scales. An additional 64 items commenced with ‘if yes’ or ‘if no’ and were only presented to a subset depending on their response to the previous item. No participant completed all items, rather there were distinct sections for management, direct support professionals, and family participants, each of which comprised approximately 70 items. These items were combined for data analysis to permit comparisons among different respondent groups. While all participants completed a demographic section, and the two wellbeing measures, which collectively comprised 23 core items, additional sections completed only by direct support professionals and family caregivers included those on information and training, experiences of COVID-19, and social distancing, which collectively comprised 34 core items. The survey is included as extended data ( Linehan *et al.,* 2022).

The survey was drafted by the Principal Investigator and further developed using an iterative process with co-investigators. Pre-testing of technical aspects of the online survey was supported by input from the Chief Technical Officer at University College Dublin. The final survey was translated from English into 14 languages and reverse-translated to English for validation by each partner who was, or had access to a colleague who was, fluent in both English and their host language ( Van de Vijver & Hambleton, 1996). The survey was available in Brazilian Portuguese, Czech, Dutch, English, French Canadian, German, Greek, Hebrew, Hindi, Italian, Mandarin, Norwegian, Nyanja, Spanish and Sweden. As per the funders’ requirements, no formal pilot was undertaken on the basis that this research was to be expedited, the research group had considerable experience and expertise in disability, the research group had access to specialists in online surveys, and the survey was to be kept as simple as possible to accommodate translation into multiple languages. For these reasons, and in keeping with the study’s ethical approval, each co-investigator was asked to pilot the survey *via* their personal networks with one to two individual caregivers to determine the appropriateness of the format and length of the survey. The survey content was found to be acceptable to pilot participants and no changes were warranted.

### (4) Recruitment process and description of the sample having access to the survey

The study co-investigators were provided with a template text for websites and social media announcements, specifically Twitter and Facebook, to create awareness of the survey in their country. This was accompanied by a link to the survey. Co-investigators were asked to create a list of disability and advocacy organisations in their countries to whom the text would be circulated and to create as much awareness as possible using a snowball approach where those who received information about the study were asked to share onward. An example of a tweet was “If you are a family member/paid caregiver of individuals with intellectual and developmental disabilities, help us understand their needs during Covid.” The inclusion criteria required participants to be 18 years or older and a caregiver to a person with an IDD. Once participants confirmed they met the inclusion criteria and provided informed consent, they progressed through the survey section by section (non-randomised). Participants were required to provide an answer for key questions, typically those which commenced a section, but given the quantum of adaptive questions (‘if yes’ or ‘if no’) were not forced to answer all items to progress through the survey.

### (5) Survey administration

The survey was hosted by the online survey platform Qualtrics Core XMTM ( https://www.qualtrics.com/). The survey was classified as ‘open’ (no password requirements) using a convenience sample. Participants were informed that the survey was voluntary, without incentives, and that they could exit at any point. The survey was live for 38 days from August 22 ^nd^ to September 28 ^th^, 2020. The minimum duration the survey was open across all countries was 26 days. While CHERRIES requests an indication of the number of questions presented per page, this is not possible to address as the survey presentation differed markedly depending on the device used by participants, such as mobile phone, iPad, or laptop. CHERRIES also requests information on the number of individuals identified on the ‘landing’ page of the survey. In fact, this survey did not have a conventional landing page, as had been anticipated in the study protocol, rather participants were directly brought to the survey on Qualtrics.

### (6) Response rates

**Figure 1.**
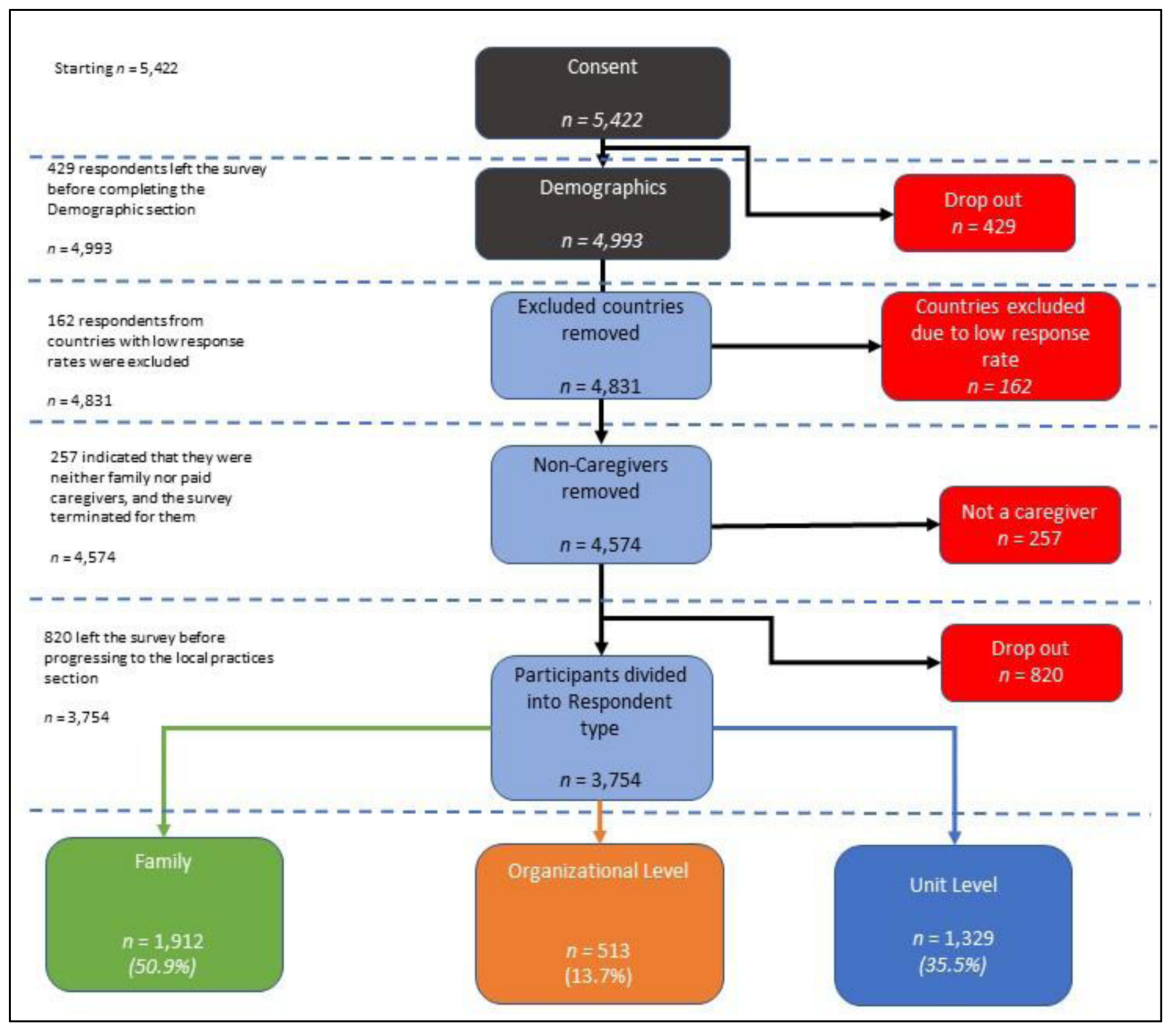
Attrition rate of survey from consent through to final sample used in data analysis.

CHERRIES defines response rates as the ratio of unique visitors who consented to participate divided by the number of individuals who visited the first page of the survey. The ratio of the number of persons who opened the survey link to the number who visited the first page of the survey was 0.92. CHERRIES defines the completion rate as the number who completed the last page of the survey divided by the number who consented to participate, a ratio of 0.63 in this survey. In total 5,422 individuals completed the consent section of the survey. Figure 1 illustrates the attrition of responses from consent through to data deemed appropriate for inclusion in data analysis. A total of 429 individuals were excluded for quitting the survey before completing the demographic section. A further 162 participants were excluded from data analysis, with the agreement of co-investigators, on the basis that they were submitted from countries with very low response rates (n=<29) and would likely produce within cell data of less than five which raised issues regarding anonymity. For this reason, respondents from the following six countries were excluded from analyses: Brazil, Czech Republic, Germany, Greece, Portugal, and Zambia. Consequently, the following countries were included in the data analysis: Australia, Canada, Hong Kong SAR, India, Ireland, Israel, Italy, the Netherlands, Norway, Sweden, UK, and US. An additional 257 respondents were excluded on the basis that they did not identify as either a family member or a paid staff within disability services, and consequently the survey was terminated. Finally, 820 responses were excluded as the respondents quit the survey before the first section.

### Participants

As Figure 1 illustrates, the final sample size was n=3,754; 1,912 family members, 1,329 direct support professionals, and 513 managers. Participants were asked to self-classify to just one of these categories for the purpose of the study, mindful that some family members may also be employees in disability organisations. Table 1 presents the number of participants by respondent type from all 12 countries. Table 2 and Table 3 present demographic information for participants and information on the size of participating organisations. 

**Table 1.** Number (n) and percentage (%) of participants by respondent type and country.

Respondent type	Family members		Direct support professionals		Managers		Total	
	n	%	n	%	n	%	n	%
**Total**	1,912	50.9%	1,329	35.4%	513	13.7%	3,754	100.0%
**Country**								
Sweden	340	17.8%	507	38.1%	72	14.0%	919	24.5%
Netherlands	255	13.3%	209	15.7%	30	5.8%	494	13.2%
Canada	261	13.7%	110	8.3%	88	17.2%	459	12.2%
USA	152	7.9%	58	4.4%	72	14.0%	282	7.5%
Hong Kong SAR	208	10.9%	47	3.5%	14	2.7%	269	7.2%
India	98	5.1%	77	5.8%	81	15.8%	256	6.8%
Ireland	167	8.7%	51	3.8%	35	6.8%	253	6.7%
Norway	92	4.8%	116	8.7%	11	2.1%	219	5.8%
Italy	106	5.5%	76	5.7%	22	4.3%	204	5.4%
Israel	75	3.9%	36	2.7%	49	9.6%	160	4.3%
Australia	78	4.1%	21	1.6%	28	5.5%	127	3.4%
UK	80	4.2%	21	1.6%	11	2.1%	112	3.0%

**Table 2.** Participant demographics by respondent type.

	Family members			Direct support professionals			Managers			Total		
	Total ^1^	Yes ^2^	%	Total ^1^	Yes ^2^	%	Total ^1^	Yes ^2^	%	Total ^1^	Yes ^2^	%
Age of caregiver	1,912		100%	1,329		100%	513		100%	3,754		100%
18 to 34 years		173	9.0%		366	27.5%		84	16.4%		623	16.6%
35 to 49 years		595	31.2%		476	35.8%		208	40.5%		1,279	34.0%
50 to 64 years		784	41.0%		466	35.1%		198	38.6%		1,448	38.6%
65+ years		360	18.8%		21	1.6%		23	4.5%		404	10.8%
Gender of caregiver	1,883		100%	1,307		100%	505		100%	3,695		100%
Male		334	17.7%		225	17.2%		126	25.0%		685	18.5%
Female		1,549	82.3%		1,082	82.8%		379	75.0%		3,010	81.5%

^1^ Total number of respondents who answered the survey item. ^2^ Total number of respondents who positively endorsed each response option.

**Table 3.** Size of disability organisation as reported by direct support professionals and managers.

	Direct support professionals			Managers			Total		
	Total ^1^	Yes ^2^	%	Total ^1^	Yes ^2^	%	Total ^1^	Yes ^2^	%
Staff organisations as profiled by the number of persons they support	1,329		100%	513		100%	1,842		100%
More than 300		554	41.7%		220	42.9%		774	42.0%
100 - 299		276	20.8%		135	26.3%		411	22.3%
Less than 100		499	37.5%		158	30.8%		657	35.7%
Staff organisations as profiled by the number of staff they employ	1,324		100%	513		100%	1,837		100%
More than 100		818	61.8%		306	59.6%		1,124	61.2%
50 to 100		162	12.2%		83	16.2%		245	13.3%
Less than 50		344	26.0%		124	24.2%		468	25.5%

^1^ Total number of respondents who answered the survey item. ^2^ Total number of respondents who positively endorsed each response option.

### (7) Preventing multiple entries from the same individual

To support the anonymous nature of the survey, no computer IP addresses were recorded during data collection. Consequently, it is not possible to determine if participants submitted more than one survey. In fact, even if IP addresses had been captured, a participant could have made multiple responses using different computers. While the risk of multiple entries from the same individual exists, it is outweighed by the need for anonymity and is deemed unlikely given the nature of the survey.

### (8) Data analysis

Data were analysed using IBM SPSS Statistics (Version 26) ( https://www.ibm.com › analytics › spss-statistics-software). The following steps were taken to prepare data for analysis. ‘Not applicable’ responses were recoded to missing values where appropriate to allow valid percentages to be ascertained and binary variables to be created for entry into regression analyses.

Three additional variables were added to the dataset to control for firstly, severity of COVID-19 and secondly, level of restrictions imposed within each country. These variables were deemed to be more appropriate for inclusion in regression analyses than the sole variable ‘country’, gathered from the survey, which failed to acknowledge levels of severity and restrictions. Two indicators were taken from the Our World in Data website ( https://ourworldindata.org/) for the exact period of data collection in each country (1) the number of cases of COVID-19 per million and (2) the total number of COVID-19 deaths per million. The third variable was an indicator of the severity of restrictions within each country for the data collection period, titled the ‘Stringency Index’ developed by Oxford University and Blavatnik School of Government ( https://www.bsg.ox.ac.uk/research/research-projects/covid-19-government-response-tracker).

The dataset is archived at Open Science Framework, details of which are presented in the data availability section below. ( Linehan *et al.,* 2022).

## Results

These results are based on responses from 3,754 caregivers from 12 countries of whom 1,912 identified as family members, 1,329 as direct support professionals, and 513 as managers (see Table 1).

Results are presented in three sections. Firstly, descriptive tables outline family members’ and paid staff’s perceptions of the impact of the COVID-19 pandemic on individuals with IDD and their caregivers. Secondly, factors which impact on the wellbeing of these caregivers and the individuals they support during the pandemic were explored using regression analyses. Finally, differences in the self-reported experiences of participants supporting individuals with IDD who lived in different living arrangements were also explored. As discussed in some detail in the published study protocol for this research ( Linehan *et al.,* 2020), this study is limited by reporting on the perceptions of caregivers and does not include the direct voice of people with IDD. Given this limitation, the Principal Investigator extended an invitation to Inclusion International ( https://inclusion-international.org/) to present a set of preliminary findings via Zoom ( https://zoom.us/) meetings to a group of self-advocates and direct support professionals. This was not outlined in the methods as it was a *post-hoc* discussion and not included in the original process. Following two zoom discussions, the findings were found to generally resonate with the lived experience of these advocates and staff.

### Perception of family members and paid staff on the impact of the COVID-19 pandemic

The profile of persons with IDD who were supported by family members and direct support professionals is outlined in terms of gender, age, living arrangement, and level of paid support ( Table 4). The table also includes ‘characteristics of the person with IDD’ where participants were presented with a list of characteristics such as intellectual disability, difficulty with self-care, and epilepsy. It’s important to note that participants were asked to endorse all relevant items and consequently the same individual may be represented in multiple items in this section. As Table 4 reveals, higher proportions of direct support professionals, when compared with family members, supported individuals with specific disabilities, such as autism and epilepsy, and who had specific difficulties such as communication and self-care. Paid staff were also more likely than family members to support individuals with IDD who received 24 hour paid support and lived outside of the family home. 

**Table 4.** Demographics and characteristics of the person(s) supported by family members and direct support professionals.

	Family members (n=1,912)			Direct support professionals (n=1,329)			Total (n=3,241)		
	Total ^1^	Yes ^2^	%	Total ^1^	Yes ^2^	%	Total ^1^	Yes ^2^	%
Gender of person(s) with IDD supported by caregivers	1,906		100%	1,288		100%	3,194		100%
Male		1,135	59.5%		132	10.2%		1,267	39.7%
Female		771	40.5%		78	6.1%		849	26.6%
Group of males and females supported		0	0.0%		1,078	83.7%		1,078	33.8%
Age group of person(s) with IDD supported by caregivers	1,911		100%	1,285		100%	3,196		100%
Child/group of children		530	27.7%		93	7.2%		623	19.5%
Adult/group of adults		1,381	72.3%		1,093	85.1%		2,474	77.4%
Group of persons being supported including adults and children		0	0.0%		99	7.7%		99	3.1%
Characteristics of person(s) with IDD supported by caregivers	1,906		100%	1,323		100%	3,229		100%
Intellectual disability		1,655	86.8%		1,290	97.5%		2,945	91.2%
Difficulty with self-care such as washing or dressing		1,015	53.3%		991	74.9%		2,006	62.1%
Difficulty communicating, understanding or being understood		1,002	52.6%		962	72.7%		1,964	60.8%
Autism spectrum disorder		798	41.9%		1,050	79.4%		1,848	57.2%
Epilepsy		424	22.2%		784	59.3%		1,208	37.4%
Challenging Behaviour		404	21.2%		690	52.2%		1,094	33.9%
Living arrangements of person(s) with IDD	1,907		100%	1,315		100%	3,222		100%
Family Home		1,164	61.0%		65	4.9%		1,229	38.1%
Community Group Home		341	17.9%		501	38.1%		842	26.1%
Living in more than one setting		117	6.1%		349	26.5%		466	14.5%
Residential center		168	8.8%		235	17.9%		403	12.5%
Independent Living		99	5.2%		148	11.3%		247	7.7%
Other		18	0.9%		17	1.3%		35	1.1%
Levels of paid support for person(s) with IDD									
Number with 24-hour paid support	1,904	670	35.2%	1,324	913	69.0%	3,228	1,583	49.0%
< 24-hour paid support, *with some paid support* ^3^	1,187	524	44.1%	140	125	89.3%	1,327	649	48.9%
< 24-hour paid support, *with no paid support* ^3^	1,187	663	55.9%	140	15	10.7%	1,327	678	51.1%

^1^ Total number of respondents who answered the survey item. ^2^ Total number of respondents who positively endorsed each response option. ^3^Only presented to those who responded ‘no’ to supporting a person with 24 hour paid staff.

The observed impact of the pandemic on individuals with IDD is presented in Table 5 as reported by family members and direct support professionals. These observations include increases in challenging behaviour for those who engaged in these behaviours pre-pandemic, changes in mood, increased repetitive behaviours, increased screen time, reduced physical activity, and reduced contact with social support networks, notably visits to and from family and friends. Over 40% of families reported that they avoided supporting their family member with IDD to attend healthcare facilities due to the pandemic. Difficulties getting prescriptions were experienced by approximately 15%-20% of caregivers, with families reporting greater levels of difficulty than paid staff. Almost one in three caregivers observed increased use of psychotropic medication among the people they supported.

**Table 5.** Observed impact of COVID-19 pandemic on person(s) with intellectual and developmental disability as reported by family members and direct support professionals.

	Family members (n=1,912)			Direct support professionals (n=1,329)			Total (n=3,241)		
	Total ^1^	Yes ^2^	%	Total ^1^	Yes ^2^	%	Total ^1^	Yes ^2^	%
Changes observed in person(s) with IDD during the pandemic									
More screen time than usual	1,392	1,125	80.8%	963	682	70.8%	2,355	1,807	76.7%
More changes in mood (depression, anxiety) than usual	1,313	854	65.0%	1,080	678	62.8%	2,393	1,532	64.0%
More repetitive/stereotyped behaviours than usual	1,224	680	55.6%	978	434	44.4%	2,202	1,114	50.6%
More aggressive behaviours than usual towards others	860	424	49.3%	953	427	44.8%	1,813	851	46.9%
More weight gain than usual	1,357	585	43.1%	1,023	459	44.9%	2,380	1,044	43.9%
More self-harm than usual	527	244	46.3%	723	241	33.3%	1,250	485	38.8%
More sleep problems than usual	1,395	495	35.5%	975	314	32.2%	2,370	809	34.1%
More use of psychotropic medication for mood or behaviour	575	176	30.6%	822	232	28.2%	1,397	408	29.2%
Less contact than usual with their social support network	1,501	1,189	79.2%	1,061	652	61.5%	2,562	1,841	71.9%
Less physical activity than usual	1,692	1,068	63.1%	1,176	583	49.6%	2,868	1,651	57.6%
Less exposure to sunshine than usual	1,589	770	48.5%	1,074	404	37.6%	2,663	1,174	44.1%
Increase in number of seizures for *those with epilepsy*	423	86	20.3%	782	119	15.2%	1,205	205	17.0%
Increase in challenging behaviour for *those with pre-* *existing behaviours that challenge*	403	268	66.5%	685	416	60.7%	1,088	684	62.9%
Restrictions to social support									
Visits to and from family restricted	1,736	1,422	81.9%	1,212	1,016	83.8%	2,948	2,438	82.7%
Visits to and from friends restricted	1,646	1,349	82.0%	1,200	1,002	83.5%	2,846	2,351	82.6%
Use of health services & COVID-19 symptoms									
Difficulties in getting prescribed anti-seizure medication	406	97	23.9%	561	68	12.1%	967	165	17.1%
Difficulties in getting prescribed psychotropic medication	410	76	18.5%	644	81	12.6%	1,054	157	14.9%
Difficulties in getting medication prescribed for other reasons	759	203	26.7%	727	127	17.5%	1,486	330	22.2%
Family carer avoided supporting family members to attend healthcare facilities	1,487	620	41.7%		-	-	1,487	620	41.7%

^1^ Total number of respondents who answered the survey item. ^2^ Total number of respondents who positively endorsed each response option.

Table 6 reports on incidents of exploitation and abuse against persons with IDD as observed by family members and direct support professionals during the pandemic, a time when individuals with IDD may have been more socially isolated and therefore more vulnerable to abuse. Caregiver reports of money/possessions being taken during the pandemic, or more seriously of physical or sexual abuse, were reported by 2–3% of caregivers. The proportion of staff who stated that they knew who to report these incidents to, and who reported all such incidents, was higher than that reported by family members. Incidents of neglect were reported by 8% of caregivers, with a similar pattern of staff being more likely than family members to know who to report these incidents of neglect to, and to report all such incidents. Almost one in five staff were aware of an increase in the use of physical restraint, and more than one in two staff were aware of an increase in environmental restraint; higher figures than those reported by family.

**Table 6.** Family members and direct support professional reports of incidents of exploitation against persons with intellectual and developmental disabilities (IDD) during COVID-19 pandemic.

	Family members (n=1,912)			Direct support professionals (n=1,329)			Total (n=3,241)		
	Total ^1^	Yes ^2^	%	Total ^1^	Yes ^2^	%	Total ^1^	Yes ^2^	%
Incidents of money or possessions taken during pandemic	1,727	57	3.3%	1,208	34	2.8%	2,935	91	3.1%
*If yes,* did caregivers know who to report incidents to?	53	22	41.5%	34	33	97.1%	87	55	63.2%
*If yes,* did caregivers report *all* of these incidents?	53	12	22.6%	34	22	64.7%	87	34	39.1%
Incidents of physical or sexual abuse during pandemic	1,746	38	2.2%	1,212	38	3.1%	2,958	76	2.6%
*If yes,* did caregivers know who to report incidents to?	38	28	73.7%	38	35	92.1%	76	63	82.9%
*If yes,* did caregivers report *all* of these incidents?	37	20	54.1%	38	30	78.9%	75	50	66.7%
Incidents of neglect during pandemic	1,746	142	8.1%	1,213	91	7.5%	2,959	233	7.9%
*If yes,* did caregivers know who to report incidents to?	141	88	62.4%	90	84	93.3%	231	172	74.5%
*If yes,* did caregivers report *all* of these incidents?	141	34	24.1%	89	50	56.2%	230	84	36.5%
Incidents of restraint									
Incidents of physical restraint ^3^ during pandemic	1,743	226	13.0%	1,211	232	19.2%	2,954	458	15.5%
Incidents of environmental restraint ^4^ during pandemic	1,740	648	37.2%	1,211	683	56.4%	2,951	1,331	45.1%

^1^ Total number of respondents who answered the survey item. ^2^ Total number of respondents who gave a positive response ‘yes’ to the survey item. ^3 ^ Physical restraint was defined as any manual method or physical or mechanical device, material or equipment attached or adjacent to the person’s body that the individual cannot easily remove that restricts freedom of movement or normal access to one’s body. ^4^ Environmental restraint was defined as intentional restriction of a person’s normal access to their environment, with the intention of stopping them from leaving, or denying a person their normal means of independent mobility, means of communication or intentional taking away of ability to exercise civil and religious liberties.

The experiences of both caregivers and the people they support becoming symptomatic, diagnosed, and/or treated for COVID-19 are presented in Table 7. Over one in four family members and direct support professionals reported that the person they supported exhibited COVID-19 symptoms, with direct support professionals three times more likely to report this observation than family. The majority of symptomatic individuals were quarantined. Almost one third of supported persons were tested, including routine testing, and of these, almost one quarter were diagnosed with COVID-19. Of those diagnosed, over one quarter were hospitalised. The hospital experiences are reported in Table 7 however the numbers are small and therefore should be treated with caution. Mindful of this caution, the data reveals a trend whereby direct support professionals were more likely than family to report symptoms, quarantining, testing and diagnosis among the persons they support, yet both caregiver groups reported similar rates of hospitalisation. Approximately 8% of caregivers of persons with IDD who exhibited symptoms felt that the person they supported was refused treatment for COVID-19 due to their disability status. In total, 27 caregivers reported that the person they supported died from COVID-19.

**Table 7.** Experience of COVID-19 as reported by family members and direct support professionals.

Experiences of COVID-19 among supported persons with intellectual and developmental disability							Experiences of COVID-19 among family members and direct support professionals						
	Family members (n=1,192)		Direct support professional (n=1,329)		Total (n=3,241)			Family members (n=1,192)		Direct support professional (n=1,329)		Total (n=3,241)	
	Yes ^1^	%	Yes ^1^	%	Yes ^1^	%		Yes ^1^	%	Yes ^1^	%	Yes ^1^	%
Did person show symptoms?	202	11.4%	482	38.3%	684	22.5%	Did you show symptoms?	240	13.5%	317	25.1%	557	18.3%
*If yes,* were they quarantined?	138	69.3%	420	88.1%	558	82.5%	*If yes,* were you quarantined?	137	57.1%	248	78.2%	385	69.1%
Was person(s) tested?	356	20.1%	605	50.8%	961	32.4%	Did you get tested?	377	21.3%	215	67.8%	592	28.4%
*If yes,* were they diagnosed?	56	15.8%	180	30.1%	236	24.8%	*If yes,* were you diagnosed?	37	15.5%	37	11.7%	74	13.4%
*If yes,* were they hospitalised?	15	26.8%	49	27.2%	64	27.1%	*If yes*, were you hospitalised?	9	3.8%	5	1.6%	14	2.5%
*If person was hospitalised:*							*If you were hospitalised:*						
Could someone stay with the person in hospital?							Were you treated in ICU ^2^	<10	-	<5	-	<10	-
*Family member*	5	33.3%	8	16.3%	13	20.3%							
*Paid staff member ^2^*	<5	-	<10	-	<15	-							
Did hospital receive support information (health passport)	8	53.3%	31	81.6%	39	73.6%	Who provided support during your illness?						
Do you think symptomatic:							*Family member*	123	65.8%	13	4.7%	136	29.5%
person(s) was							*Paid staff member*	61	32.6%	261	100.0%	322	71.9%
*Refused access to ICU for* *COVID-19 due to disability ^2^*	<5	-	<5	-	<10	-	*Other*	34	18.2%	25	8.7%	59	12.5%
*Refused treatment for* *COVID-19 due to disability*	18	8.9%	36	7.6%	54	8.0%							
Did the person(s) you support die due to COVID-19?	0	-	27	-	27	-							

^1^Total number of respondents who gave a positive response ‘yes’ to the survey item. ^2^In cells with less than five respondents we have used ‘<5’ in keeping with good practice in data presentation: where this number could be identified due to allied data, these allied data are denoted as <10/<15.

Almost one in four family members and direct support professionals reported exhibiting symptoms, with two thirds being quarantined. Over a quarter were tested, including routine testing, with testing rates among paid staff being three times higher than among family members. Diagnoses and hospitalisation rates among caregivers were low and should be interpreted with caution. Where caregivers became ill with COVID-19, family members most typically relied on other family members to provide support while paid staff typically relied on other paid staff.

The impact of the pandemic on family members supporting individuals with IDD during the pandemic is outlined in Table 8. Almost two-thirds of family members avoided healthcare facilities, almost one in five stopped work due to their caregiving duties, and over a third reduced working hours. Over half reported spending more money on the person they supported, and in cases where the person with IDD had a personal budget, almost one in four stated it was negatively impacted by the pandemic. The majority of family members were dissatisfied with the levels of support they and their family members with IDD received during the pandemic.

**Table 8.** Self-reported impact of COVID-19 pandemic by family members.

	Family members (n=1,912)		
	Total ^1^	Yes ^2^	%
Accessing healthcare and shopping			
Family carer avoided attending healthcare facilities due to pandemic	1,652	1,048	63.4%
Family experienced difficulty shopping for food, medicines or hygiene products	1,708	664	38.9%
Employment / income			
Were you employed before the COVID-19 pandemic?	1,657	1,062	64.1%
Did you become unemployed during the COVID-19 pandemic?	1,057	181	17.1%
Did you stop working because you needed to support your family member?	1,055	203	19.2%
Did you have to reduce the hours that you normally go to work because you needed to support your family member?	1,051	379	36.1%
Did you work from home during the COVID-19 pandemic	1,053	611	58.0%
Did your income become reduced directly because of the COVID-19 pandemic?	1,613	515	31.9%
Did you spend more money on your family member to meet their needs than you usually do?	1,729	910	52.6%
Does your family member receive a personal budget (also termed an individual payment)?	1,628	880	54.1%
*If yes,* was the personal budget negatively impacted by additional levies or purchases?	875	209	23.9%
Positive satisfaction with support			
Those satisfied with the level of support family member received during pandemic	1,816	604	33.3%
Those satisfied with the level of support they received in caregiving role during pandemic	1,815	456	25.1%

^1^ Total number of respondents who answered the survey item. ^2^ Total number of respondents who positively endorsed each response option.

The impact of the pandemic on service provision is presented in Table 9. Cancellations or reductions of services were widely reported with high rates observed for core supports such as day programmes, social, exercise and educational activities. Less than half of respondents reported that alternative services were developed to replace cancelled or reduced services, with direct support professionals and management almost twice as likely as family to report alternative services. Over half of family caregivers expressed concern about how their family member would respond to a return to these services.

**Table 9.** Family and paid staff reporting of cancellation or reduction in services and activities during COVID-19.

	Family members (n=1,912)			All paid staff (n=1,842)			Total (n=3,754)		
	Total ^1^	Yes ^2^	%	Total ^1^	Yes ^2^	%	Total ^1^	Yes ^2^	%
Cancellations or reductions among those using structured programmes									
Day Programmes	1,281	1,067	83.3%	1,677	1,438	85.7%	2,958	2,505	84.7%
Respite Services	724	481	66.4%	1,110	610	55.0%	1,834	1,091	59.5%
Home Care Services	525	290	55.2%	1,013	401	39.6%	1,538	691	44.9%
Cancellations or reductions among those availing of social and faith-based activities									
Group Social Activities	1,277	1,117	87.5%	1,631	1,479	90.7%	2,908	2,596	89.3%
Individual Social Activities	1,412	1,242	88.0%	1,637	1,385	84.6%	3,049	2,627	86.2%
Faith based activities	560	382	68.2%	951	566	59.5%	1,511	948	62.7%
Cancellations or reductions among those availing of exercise activities									
Group Exercise Activities	1,121	981	87.5%	1,509	1,360	90.1%	2,630	2,341	89.0%
Individual Exercise Activities	1,227	1,076	87.7%	1,544	1,334	86.4%	2,771	2,410	87.0%
Cancellations or reductions among those availing of educational and/or employment services									
Group educational activities	653	489	74.9%	1,280	1,019	79.6%	1,933	1,508	78.0%
Individual educational activities	696	525	75.4%	1,286	973	75.7%	1,982	1,498	75.6%
Sheltered workshops	582	392	67.4%	1,203	886	73.6%	1,785	1,278	71.6%
Support services to gain employment	513	315	61.4%	1,240	804	64.8%	1,753	1,119	63.8%
Special schools	549	360	65.6%	857	489	57.1%	1,406	849	60.4%
Mainstream schools	456	292	64.0%	714	380	53.2%	1,170	672	57.4%
Introduction of new/alternative supports to replace services that were cancelled or reduced	1,707	590	34.6%	1,692	1,100	65.0%	3,399	1,690	49.7%
Family respondents who were concerned about how family member would respond to a return to these services	1,636	943	57.6%	-	-	-	1,636	943	57.6%

^1^ Total number of respondents who answered the survey item. ^2^ Total number of respondents who gave a positive response ‘yes’ to the survey item.

Adjustments to paid staff support during the pandemic are reported in Table 10. Half of all respondents, including family, direct support professionals and management, reported that staff shifts were reorganized to reduce social contact. One in four observed an increase in new staff, of whom approximately half were observed to be casual staff. Almost half of all participants observed an increase in staff sick leave. One in two management and direct support professionals reported staff taking on additional tasks, of which only half were financially reimbursed.

**Table 10.** Adjustments to paid staff supports during COVID-19.

	Family members (n=1,912)			All paid staff (n=1,842)			Total (n=3,754)		
	Total ^1^	Yes ^2^	%	Total ^1^	Yes ^2^	%	Total ^1^	Yes ^2^	%
Staffing issues									
Staff shifts reorganised to reduce contact with person(s)	847	481	56.8%	1,615	929	57.5%	2,642	1,410	57.3%
Increase in new direct support staff	1,061	268	25.3%	1,763	492	27.9%	2,824	760	26.9%
*If yes,* increase in casual new staff	168	114	67.9%	411	199	48.4%	579	313	54.1%
Increase in staff on sick leave	1,037	345	33.3%	1,789	959	53.6%	2,826	1,304	46.1%
Holiday leave reduced/cancelled	-	-	-	1,773	430	24.3%	1,773	430	24.3%
Staff asked to take holiday leave if unable to attend work	-	-	-	1,735	387	22.3%	1,735	387	22.3%
Increased workload/number of shifts	-	-	-	1,744	695	39.9%	1,744	695	39.9%
Staff asked to take on additional tasks	-	-	-	1,788	925	52.0%	1,788	925	52.0%
Staff paid for additional tasks or shifts	-	-	-	1,562	855	54.7%	1,562	855	54.7%
Staff asked to live apart from their own families	-	-	-	1,733	127	7.3%	1,733	127	7.3%
*If yes,* staff asked to live with people they support in a residential setting	-	-	-	119	69	58.0%	119	69	58.0%

^1^ Total number of respondents who answered the survey item. ^2^ Total number of respondents who gave a positive response ‘yes’ to the survey item.

The use of online supports, such as mobile phones, email, Zoom, and Whatsapp ( https://www.whatsapp.com/), was adopted by three quarters of all respondents. While over half had used online supports prior to the pandemic, four out of five respondents reported increased usage during the pandemic. A minority reported internet difficulties and receiving funding for these supports (see Table 11).

**Table 11.** How family members and paid staff supported communication for persons with IDD with their family and friends.

	Family members (n=1,912)			All paid staff (n=1,842)			Total (n=3,754)		
	Total ^1^	Yes ^2^	%	Total ^1^	Yes ^2^	%	Total ^1^	Yes ^2^	%
How caregivers supported communication with family and friends									
Did caregivers use online tools (e.g., phones, email, Zoom, Whatsapp) to support people with IDD communicate with friends and family?	1,631	1183	72.5%	1,697	1,346	79.3%	3,328	2,529	76.0%
*If yes*, was this type of online communication used before COVID-19?	1,175	713	60.7%	1,338	721	53.9%	2,513	1,434	57.1%
*If yes*, was this type of communication used more than before pandemic?	712	519	72.9%	970	815	84.0%	1,682	1,334	79.3%
Did caregivers experience difficulty with internet during the pandemic?	1,676	332	19.8%	1,807	378	20.9%	3,483	710	20.4%
Was funding was made available for communication devices (e.g., iPads)?	1,629	148	9.1%	1,707	333	19.5%	3,336	481	14.4%

^1^ Total number of respondents who answered the survey item. ^2^ Total number of respondents who gave a positive response ‘yes’ to the survey item.

The distribution of policy/guidelines on COVID-19 for caregivers and easy to read versions for persons with IDD are reported in Table 12. The availability of these resources was markedly higher for paid staff when compared to family members. Whereas three quarters of staff reported satisfaction with the guidelines they received, family members reported lower levels of satisfaction.

**Table 12.** Access to COVID-19 policies and guidelines for family members and paid staff.

	Family members (n=1,912)			All paid staff (n=1,842)			Total (n=3,754)		
	Total ^1^	Yes ^2^	%	Total ^1^	Yes ^2^	%	Total ^1^	Yes ^2^	%
Caregivers access to policies/guidelines									
Did caregivers receive policy/guidelines on COVID-19 for people with IDD?	1,907	765	40.1%	1,835	1,748	95.3%	3,742	2,513	67.2%
*If yes,* proportion reporting satisfaction with the policy or guidelines	760	441	58.0%	1,740	1,351	77.6%	2,500	1,792	71.7%
Did caregivers receive easy-to-read policy/ guidelines for people with IDD?	1,903	477	25.1%	1,773	1,155	65.0%	3,676	1,632	44.4%

^1^ Total number of respondents who answered the survey item. ^2^ Total number of respondents who gave a positive response ‘yes’ to the survey item

Access to information and training for those in direct contact with individuals with IDD, that is family members and direct support professionals, is reported is Table 13. While both groups reported high rates of access to information and training on social distancing and prevention, a trend is evident favouring direct support professionals receiving information and training across all items when compared with family members. Employers were the top source of information for direct support professionals, while the internet was the most common source for family. Direct support professionals reported higher levels of satisfaction than family with both the timing and standard of information they received.

**Table 13.** Access to information and training for family members and direct support professionals.

	Family members (n=1,912)			Direct support professionals (n=1,329)			Total (n=3,241)		
	Total ^1^	Yes ^2^	%	Total ^1^	Yes ^2^	%	Total ^1^	Yes ^2^	%
Caregivers access to information and/or training in the following areas:									
Social distancing	1,322	1,108	83.8%	1,246	1,093	87.7%	2,568	2,201	85.7%
Preventing COVID-19 (infection control)	1,322	973	73.6%	1,246	1,095	87.9%	2,568	2,068	80.5%
Using Personal Protective Equipment (PPE)	1,322	717	54.2%	1,246	1,083	86.9%	2,568	1,800	70.1%
Managing symptoms of COVID-19	1,322	752	56.9%	1,246	975	78.3%	2,568	1,727	67.3%
Isolating someone who has or is suspected of having COVID-19	1,322	604	45.7%	1,246	892	71.6%	2,568	1,496	58.3%
Accessible information on the pandemic for people with IDD	1,322	419	31.7%	1,246	738	59.2%	2,568	1,157	45.1%
Contact information for support groups and/or helplines	1,322	351	26.6%	1,246	530	42.5%	2,568	881	34.3%
How information was delivered to caregivers									
Internet	1,316	898	68.2%	1,245	882	70.8%	2,561	1,780	69.5%
Government communication	1,316	795	60.4%	1,245	797	64.0%	2,561	1,592	62.2%
TV	1,316	874	66.4%	1,245	570	45.8%	2,561	1,444	56.4%
Employer	1,316	309	23.5%	1,245	1,092	87.7%	2,561	1,401	54.7%
Radio	1,316	360	27.4%	1,245	247	19.8%	2,561	607	23.7%
Family	1,316	306	23.3%	1,245	137	11.0%	2,561	443	17.3%
Other	1,316	276	21.0%	1,245	164	13.2%	2,561	440	17.2%
Caregivers’ satisfaction with information									
Satisfaction with the standard of the information received	1,707	893	52.3%	1,282	1,006	78.5%	2,989	1,899	63.6%
Satisfaction with the timing of the information received	1,698	822	48.4%	1,276	909	71.3%	2,974	1,731	58.2%

^1^ Total number of respondents who answered the survey item. ^2^ Total number of respondents who gave a positive response ‘yes’ to the survey item.

Both management and direct support professionals were asked about their access to and satisfaction with personal protective equipment (PPE) and other COVID-related equipment. Table 14 reports these data. Approximately three quarters of respondents expressed satisfaction with the level of PPE, whereas just over half expressed satisfaction with the timing of this equipment. Isolation facilities and the introduction of mandatory ‘test and tracing’ were cited by less than half of all respondents. Over one third of respondents were aware of inspections being conducted within their organisation.

**Table 14.** Access and satisfaction within the workplace to personal protective equipment (PPE) and COVID-19 facilities during the pandemic.

	All paid staff (n=1,842)		
	Total ^1^	Yes ^2^	%
PPE availability and satisfaction			
Latex gloves	1,767	1,655	93.7%
Surgical masks	1,785	1,522	85.3%
Disposable gowns	1,708	1,295	75.8%
Hibiscrub dispensers	1,709	1,209	70.7%
Goggles	1,683	974	57.9%
Paper towel dispensers	1,656	714	43.1%
Disposable caps	1,612	683	42.4%
Air filtration machines	1,587	193	12.2%
‘Very satisfied’ or ‘satisfied’ with level of PPE availability	1,754	1,280	73.0%
‘Very satisfied’ or ‘satisfied’ with timing of PPE availability	1,750	989	56.5%
Isolation room availability and satisfaction			
Isolation room for one person	1,464	660	45.1%
Isolation ward for multiple people	1,347	436	32.4%
Isolation building	1,327	365	27.5%
‘Very satisfied’ or ‘satisfied’ with isolation facilities	1,021	641	62.8%
Mandatory testing			
Mandatory ‘test and tracing’ was introduced in organisation	1,691	956	56.5%
Ongoing monitoring of the physical health of individuals with IDD introduced	1,744	1,104	63.3%
Ongoing monitoring of the physical health of staff introduced	1,770	769	43.4%
Staff have access to medically trained staff working in the organisation	1,684	1,068	63.4%
Audits			
Internal audit or inspection of infection control activities conducted	1,750	666	38.1%
*If yes, regular audits or inspections were conducted*	653	473	72.4%

^1^ Total number of respondents who answered the survey item. ^2^ Total number of respondents who gave a positive response ‘yes’ to the item

All respondents were asked if they had received information on the psychological impact of caregiving during the pandemic (see Table 15). Although just under a third of all respondents reported receiving such information, paid staff were three times more likely as families to receive this information. Where available, families were generally satisfied, but where not indicated that they would welcome this information.

**Table 15.** Access to psychological supports during the pandemic.

	Family members (n=1,912)			All paid staff (n=1,842)		
	Total ^1^	Yes ^2^	%	Total ^1^	Yes ^2^	%
**Received information on psychological impact of caring during pandemic**	1,731	264	15.3%	1,762	826	46.9%
For family members only:						
*If yes,* did this meet your needs?	257	181	70.4%	-	-	-
*If no,* would you have welcomed psychological support during pandemic?	1335	861	64.5%	-	-	-
Was there a drop in the number of people you typically ask for support?	1807	823	45.5%	-	-	-
For paid staff only:						
Was a peer support programme introduced?	-	-	-	1,267	263	20.8%

^1^ Total number of respondents who answered the survey item. ^2^ Total number of respondents who gave a positive response ‘yes’ to the survey item.

The classification of family members, direct support professionals and managers to three subscales of the DASS12, screening for depression, anxiety, and stress respectively, are presented in Table 16. Combining ‘moderate’ and ‘severe’ responses, high levels of stress (62.6%) and depression (40.0%) were reported, less so anxiety (21.3%). Across all three subscales, family members were more likely than direct support professionals or managers to be classified within the ‘moderate’ or ‘severe’ range. The Coronavirus Anxiety Scale identified a minority of the sample scoring within the dysfunctional anxiety range, where management and family members reported higher proportions than direct support professionals. 

**Table 16.** Number (n) and percentage (%) of family members and direct support professionals classified with depression, anxiety, and/or stress as measured by the depression, anxiety, and stress scale (DASS12) ( Ang *et al.,* 2018; Osman *et al.,* 2014) and dysfunctional anxiety as measured by the Coronavirus anxiety scale (CAS) ( Lee, 2020).

	Family members (n=1,912)		Direct support professionals (n=1,329)		Managers (n=513)		Total (n=3,754)	
	n	%	n	%			n	%
DASS12 depression subscale categories	1,696	100%	1,187	100%	483	100.0%	3,366	100.0%
Normal	425	25.1%	476	40.1%	175	36.2%	1,076	32.0%
Mild	462	27.2%	335	28.2%	147	30.4%	944	28.0%
Moderate	412	24.3%	214	18.0%	94	19.5%	720	21.4%
Severe	397	23.4%	162	13.6%	67	13.9%	626	18.6%
DASS12 anxiety subscale categories	1,691	100%	1,185	100%	482	100.0%	3,358	100.0%
Normal	739	43.7%	637	53.8%	244	50.6%	1,620	48.2%
Mild	522	30.9%	342	28.9%	158	32.8%	1,022	30.4%
Moderate	245	14.5%	137	11.6%	46	9.5%	428	12.7%
Severe	185	10.9%	69	5.8%	34	7.1%	288	8.6%
DASS12 stress subscale categories	1,698	100%	1,186	100%	485	100.0%	3,369	100.0%
Normal	234	13.8%	275	23.2%	71	14.6%	580	17.2%
Mild	295	17.4%	272	22.9%	116	23.9%	683	20.3%
Moderate	517	30.4%	331	27.9%	124	25.6%	972	28.9%
Severe	652	38.4%	308	26.0%	174	35.9%	1,134	33.7%
Coronavirus anxiety scale	1,708	(100%)	1,188	(100%)	491	(100%)	3,378	(100%)
Dysfunctional anxiety	132	(7.7%)	40	(3.4%)	43	(8.8%)	215	(6.3%)

### Exploring caregiver wellbeing

Regression analyses were conducted to explore the relationship between caregiver wellbeing, as measured by DASS12, and caregivers’ reporting of various experiences during the COVID-19 pandemic. These analyses were restricted to those providing direct support to people with IDD, that is, family members and direct support professionals. A dependent variable was constructed which classified these direct caregivers into two groups, (1) those reporting moderate or severe screenings on any one of the three DASS12 subscales, depression, anxiety, or stress and (0) those reporting moderate or severe screening on none of the three DASS12 subscales. Independent variables were selected based on theoretical expectations of variables that may contribute to wellbeing. Regressions were conducted separately for family members and for paid staff on the basis that independent variables would differ for the two respondent groups. The strongest predictors of a family member being categorised within the moderate or severe range for depression, anxiety and/or stress was their observation of mood change in the person they support, and the fact that the person lived within the family home. Other significant predictors included restrictions in visits to and from family and friends and the fact that the participant expressed dissatisfaction with the level of support provided to their family member with IDD (see Table 17).

**Table 17.** Logistic regression predicting family members’ categorisation to moderate or severe depression, anxiety, or stress as measured by DASS12 ( Ang *et al.,* 2018; Osman *et al.,* 2014).

Covariates	*β*	P value	Exp ( *β*)	95% CI for Exp ( *β*)
National COVID-19 cases per million	0.01	0.000	1.1	1.0-1.0
National COVID-19 deaths per million	0.00	>0.05	1.0	0.9-1.0
National stringency index of lockdown measures	0.02	0.021	1.0	1.0-1.0
Observed increase in changes in mood of person with IDD	0.87	0.000	2.4	1.6-3.6
Observed increase in person with IDD’s repetitive behaviours	0.30	>0.05	1.3	0.9-2.1
Restrictions of visits to and from family and friends	0.54	0.048	1.7	1.0-2.9
Family member (caregiver) experienced COVID-19 symptoms	0.41	>0.05	1.5	0.8-2.7
Family member reporting satisfaction with support for person	-0.82	0.000	0.4	0.3-0.7
Person with IDD living in family/own home	0.83	0.000	2.3	1.5-3.4
Reduction/closure of individual social activities for person	0.25	>0.05	1.2	0.7-2.3

A second logistic regression, presented in Table 18 using the same dependent variable, was run using only data from direct support professionals (n=1,329). Significant predictors of a direct support professional being categorized within the moderate or severe range for depression, anxiety, and/or stress were experiencing reorganisation of staff shifts, experiencing an increase in new staff, and expressing dissatisfaction with the timing of PPE from their employer.

**Table 18.** Logistic regression predicting direct support professionals’ categorisation to moderate or severe depression, anxiety, or stress as measured by DASS12 ( Ang *et al.,* 2018; Osman *et al.,* 2014).

Covariates	*β*	P value	Exp ( *β*)	95% CI for Exp ( *β*)
National COVID-19 cases per million	0.01	0.000	1.0	1.0-1.0
National COVID-19 deaths per million	0.00	>0.05	1.0	0.9-1.0
National stringency index of lockdown measures	0.03	0.001	1.0	1.0-1.0
Restrictions or closure of individual social activity supports	0.37	>0.05	1.4	0.9-2.3
Supporting a person who has 24 hour paid support	-0.34	>0.05	0.7	0.4-1.1
Experienced reorganisation of direct support staff shifts	0.59	0.002	1.8	1.2-2.6
Experienced increase in new direct support staff	0.46	0.017	1.6	1.1-2.3
Observed increase in person with IDD’s repetitive behaviours	0.43	>0.05	1.5	0.9-2.4
Observed increase in person with IDD’s aggressive behaviours	0.36	>0.05	1.4	0.9-2.2
Satisfaction with level of PPE provided by employer	0.03	>0.05	1.0	0.6-1.6
Satisfaction with timing of PPE provided by employer	-0.70	0.001	0.5	0.3-0.8

### Exploring differences in the self-reported experiences of those supporting people living in different living arrangements

Differences in the self-reported experiences of those supporting individuals who live in different living arrangements were explored using odds ratios. These analyses include data from family members and direct support professionals (n=2,721) reporting on individuals with IDD who lived in (1) the family home or their own home, deemed ‘home’ settings and (2) community group homes or residential centres, deemed ‘service-based’ settings. Table 19 presents odds ratios for caregiver outcomes of wellbeing (DASS classification), COVID-19 experiences and access to information.

**Table 19.** Odds ratios of caregiver wellbeing and COVID-19 experiences by residential circumstances of supported person.

		Supporting person in service-based setting (n=1,245)	Supporting person in home setting (n=1,476)	Odds ratio	95% confidence interval
Caregiver wellbeing as measured by DASS12:					
Depression, moderate to severe range	Yes	35.3%	47.1%	0.6	0.5-0.7
	No	64.7%	52.9%	1.0	
Anxiety, moderate to severe range	Yes	18.0%	25.5%	0.6	0.5-0.8
	No	82.9%	74.5%	1.0	
Stress, moderate to severe range	Yes	55.9%	69.1%	0.6	0.5-0.7
	No	44.1%	30.9%	1.0	
COVID-19 experiences of caregivers:					
Tested for COVID-19	Yes	37.0%	22.4%	2.0	1.6-2.5
	No	63.0%	77.6%	1.0	
Experienced COVID-19 symptoms	Yes	21.4%	14.3%	1.6	1.3-2.0
	No	78.6%	85.7%	1.0	
Diagnosed with COVID-19	Yes	14.1%	12.9%	1.1	0.6-1.9
	No	85.9%	87.1%	1.0	
Caregiver access and satisfaction with information:					
Access to policies on Covid and IDD	Yes	83.2%	44.2%	6.2	5.2-7.5
	No	16.8%	55.8%	1.0	
Satisfaction with policies on Covid & IDD	Yes	66.6%	67.7%	0.9	0.8-1.2
	No	33.4%	32.3%	1.0	
Satisfaction with timing of information	Yes	62.3%	51.5%	1.6	1.3-1.8
	No	37.7%	48.5%	1.0	
Given psychological support information	Yes	30.3%	22.3%	1.5	1.3-1.8
	No	69.7%	77.7%	1.0	

On most wellbeing items listed above, caregivers who supported individuals with IDD living in home settings fared worse than those supporting individuals in service-based setting. Caregivers supporting individuals in service-based settings were less likely to report being moderate to severely depressed, anxious or stressed. These caregivers were more likely than those supporting an individual in a home setting to be tested for COVID-19 but were also more likely to self-report experiencing COVID-19 symptoms. Both groups reported similar risk of diagnosis. Although those supporting a person in service-based settings were six times more likely to receive policies on COVID-19 and IDD and were more satisfied with the timing of this information, both groups reported similar levels of satisfaction with the content of the policies when received. Finally, those supporting a person in service-based setting were more likely than those supporting a person in a home setting to receive information on psychological supports. 

Similar analyses, presented in Table 20, were undertaken for caregiver reports of wellbeing for individuals with IDD living in service-based and home settings. These outcomes include experiences of COVID-19 testing, symptoms and diagnosis, changes to support services, and observed wellbeing in individuals with specific needs, notably, those who engaged in behaviours that challenge, had epilepsy or experienced sleep problems prior to the pandemic. Individuals with IDD living in service-based settings were more likely to be observed showing symptoms, being tested and being diagnosed with COVID-19. These individuals were also more likely to be physically restrained, offered alternatives when services were closed, supported by new casual staff during the pandemic, and were observed as less likely to experience reorganisation of staff shifts than those living within home settings. Observations regarding decreased wellbeing of those with specific support needs were modest across home and service-based living arrangements.

**Table 20.** Odds ratios for wellbeing among those with specific needs and COVID-19 experiences by residential circumstances of supported person.

		Supporting person in service-based setting (n=1,245)	Supporting person in home setting (n=1,476)	Odds ratio	95% confidence interval
COVID-19 issues for supported persons:					
Tested for COVID-19	Yes	44.5%	19.7%	3.3	2.7-4.0
	No	55.5%	80.3%	1.0	
Experienced COVID-19 symptoms	Yes	30.8%	12.8%	3.0	2.5-3.7
	No	69.2%	87.2%	1.0	
Diagnosed with COVID-19	Yes	26.6%	15.5%	2.0	1.3-3.3
	No	73.4%	84.5%	1.0	
Changes to support services:					
Use of physical restraint	Yes	21.1%	9.2%	2.6	2.1-3.3
	No	78.9%	90.8%	1.0	
Alternative services offered	Yes	54.6%	39.8%	1.8	1.5-2.1
	No	45.4%	60.2%	1.0	
Staff shifts reorganised	Yes	44.1%	63.7%	0.5	0.4-0.6
	No	55.9%	36.3%	1.0	
New casual staff introduced	Yes	59.5%	57.7%	1.3	0.8-2.1
	No	40.5%	42.3%	1.0	
Wellbeing for specific populations:					
Increased behaviours that challenge	Yes	60.2%	64.0%	0.8	0.6-1.1
	No	39.8%	36.0%	1.0	
Increased seizures	Yes	14.5%	19.0%	0.7	0.5-1.0
	No	85.5%	81.0%	1.0	
Increased sleep problems	Yes	36.3%	41.8%	0.8	0.6-1.0
	No	63.7%	58.2%	1.0	

## Discussion

This study sought to explore globally family members’ and paid staff’s perceptions of the impact of COVID-19 on the individuals with IDD they support, and explore their own experiences as caregivers, using data from a global online survey conducted in 12 countries worldwide. Descriptively, the findings reveal the negative impact on wellbeing of both caregivers and the individuals they support during the pandemic. Many caregivers observed the person(s) they support presenting with increased depression/anxiety, stereotyped behaviours, aggression towards others, and weight gain. Families reported economic difficulties which they directly attributed to their caregiving role, where some ceased employment, and some shouldered new additional costs to support their family member. Direct support professionals experienced reorganised staff shifts, absences due to sick leave, a greater workload which was not necessarily paid, and an increase in new casual staff. Caregivers’ wellbeing, as measured by the DASS12, revealed that one in five reported moderate to severe anxiety, almost twice as many reported moderate to severe depression, and almost two in three reported moderate to severe stress. To interpret these findings in the light of existing evidence, this discussion revisits the framework of literature presented in the introduction which classified emerging literature into three categories: (1) diagnosis, risk factors and mortality; (2) access to healthcare, vaccines and potentially discriminatory practices; and (3) Self- and proxy-reported impact of COVID-19 on the mental health and social needs of people with IDD and their caregivers

### Diagnosis, risk factors, and mortality

As an online survey conducted using a convenience sample, the current study does not contribute to epidemiological data on diagnosis or mortality rates. Of particular relevance to this study, however, is the previous literature identifying risk factors for COVID-19 among persons with IDD and their caregivers. These risk factors include co-morbid conditions such as epilepsy and mental health problems, identified by Perera *et al.* (2020) as being overly represented in those with IDD who died from COVID-19. Within this study sample, one fifth were reported to have epilepsy, two thirds were observed by caregivers to present with a change of mood indicative of depression or anxiety, over half were observed to engage in increased repetitive/stereotyped behaviours, and over a third with increased rates of self-harm. These findings suggest that many of the persons with IDD supported in this study could be deemed at high risk of COVID-19 by virtue of their neurological and mental health status during the pandemic.

The shift-based nature of support has also been cited as potential risk factor for COVID-19 ( Ervin, 2021). The reporting in this study by almost half of all direct support professionals that shifts were reorganised, combined with increases in staff sick leave, in addition to the recruitment of new staff on casual contracts is a cause for concern. This concern is not only for the possibility of asymptomatic staff transmission of COVID-19 but also for the mental wellbeing of direct support professionals themselves which, in this study, was significantly impacted by the reorganisation of shift work and increases in new staff.

Some living arrangements of persons with IDD have been identified as risk factors for COVID-19, specifically congregated settings with shared spaces, typically supporting those who are more medically and/or socially compromised ( Buono *et al.,* 2021; Courtenay & Perera, 2020). Over half of all persons with IDD supported in this study were potentially supported in congregated settings, specifically those supported in residential centres, community group homes and those reported as living in more than one setting. 

In combination, many of the risk factors for COVID-19 for persons with IDD identified in previous research were observed in this study: the presence of neurological and mental health comorbidities; disruption to staff shifts and introduction of casual staff; and the continued use of congregated settings despite national policies and international human right charters advocating their closure ( United Nations General Assembly, 2006). Given that evidence existed from previous influenza epidemics of the higher risk of mortality for persons with IDD ( Cuypers *et al.,* 2020), the failure in many international jurisdictions to protect this population ( Oakley *et al.,* 2020) requires immediate attention.

### Access to healthcare, vaccines, and potentially discriminatory practices

The present study did not ask participants about vaccination as vaccines were not on offer during the period of data collection, that is, August-September 2020. This study does, however, contribute to the existing evidence on disrupted access to healthcare and discriminatory practices experienced by individuals with IDD during the pandemic.

Previous research attesting to disrupted access to healthcare ( Drum *et al.,* 2020; Jeste *et al.,* 2020; Rosencrans *et al.,* 2021) was replicated in the present study. Family members reported that they avoided supporting their family member with IDD to attend healthcare facilities during the pandemic. Difficulties were experienced in accessing prescriptions for anti-seizure medication, psychotropic medication and prescriptions for other purposes. The implications of disruption to these medications may have significant consequences. Cessation of anti-seizure medication, for example, may contribute to the increased seizure frequency during the pandemic ( Brambilla *et al.,* 2021; Trivisano *et al.,* 2020) and drug withdrawal is a risk factor for status epilepticus, which may be fatal ( Nair *et al*., 2011). It is not only those with IDD who experienced challenges in accessing healthcare, however, as the present study found that family members supporting persons with IDD also avoided healthcare. The implications of family members avoiding healthcare are also potentially serious, with consequences not only for their own health, but also for those with IDD for whom they may be the sole source of support. For both individuals with IDD and their family members, the disrupted access to healthcare may have not only immediate consequences but also long-term implications that must be considered. 

More than one in five caregivers in the present study reported that the person(s) they supported exhibited COVID-19 symptoms. This indicator was taken to be a more accurate indicator of the likelihood of COVID-19 than diagnosis or testing on the basis that access to diagnostic and testing facilities could be highly variable across participating countries and may not be a reliable indicator of true cases. While subjective, the indicator of observed symptoms is deemed equitable across participants, and although it also cannot be deemed an indicator of positive cases, it does highlight the perception of caregivers to possible infection. Of those reporting symptoms, marked disparity was observed between family members’ and direct support professionals’ observations, with family members being considerably less likely to report COVID-19 symptoms being exhibited by the person they support. Previous research suggests there may be a possible under-reporting of COVID-19 symptoms among those with IDD given their need for additional support to understand and communicate symptoms ( Sulkes, 2020). For this reason, caregivers need to be particularly vigilant in their suspicions of symptoms, especially so for those living in congregated settings. This ‘higher index of suspicion’ ( Tenenbaum *et al.,* 2021) appears in the present study to be exercised more by direct support professionals than family members and suggests that the latter may need more education and more support in identifying and responding to possible infection. The higher reporting of COVID-19 symptoms by direct support professionals should be placed within the context of their dissatisfaction with the timing of PPE by their organisation, an issue which significantly predicted their own levels of wellbeing.

Whether issues of inaccessible healthcare, failure to provide training and accommodations for testing and diagnosis, and dissatisfaction with the timing of PPE may each be deemed discriminatory is an issue that may arise within legal argument. More broadly, the continued exclusion of people with disabilities in clinical trials has yet to be addressed. Rulings from the US are currently pending on some of these issues and others may follow ( Department of Health and Human Services, 2021; Felt *et al.,* 2021). The fact that only one third of family members in the present study reported satisfaction with the support received by their family member with IDD during the pandemic may translate into legal action post-pandemic. Data from this study also indicates that health disparities, as measured in terms of equitable access and failures of accommodation, remain an issue for persons with IDD ( van Schrojenstein Lantman de Valk *et al.,* 2000; Krahn *et al*. (2006).

### Self- and proxy-reported impact of COVID-19 on the mental health and social needs of people with IDD and their caregivers

This study replicates globally what other studies have reported locally and nationally regarding the impact of the COVID-19 pandemic on caregivers and the people they support. Caregivers observed changes in mood, stereotypy, aggression, and self-harm among the people they support which may be indicative of mental health difficulties previously reported in a growing body of research evidencing the negative toil of the pandemic on persons with IDD ( Amor *et al.,* 2021; Gleason *et al.,* 2021; Navas *et al.,* 2021; Rauf *et al.,* 2021; Schuengel *et al.,* 2020; Wieting *et al.,* 2021; Zaagsma *et al.,* 2020).

Family members in the present study reported high levels of stress and depression, less so anxiety. This finding differs from previous studies which report comparable levels of depression and anxiety among family caregivers ( Willner *et al.,* 2020). These differences may reflect a true difference in findings, or may reflect the use of different measurement tools, or the influence of the inclusion of a measure of stress in the present study. Given that the findings of high levels of anxiety among family caregivers have also been reported in qualitative studies ( Embregts *et al.,* 2021) it may be that the measure used in the present study was not sufficiently sensitive to detect the anxieties expressed in previous studies. The general trend of findings in the present study of elevated rates of indicators of mental health among family caregivers is in keeping with previous research.

Similar to family members, the direct support professionals participating in this study reported elevated rates of stress, less so depression and anxiety. These findings differ from previous contrasting studies which found comparable high levels of depression and anxiety among support staff ( Lunsky *et al.,* 2021c) and findings of milder levels of anxiety and depression ( McMahon *et al.,* 2020). As noted above this disparity may be a true reflection of differences in the samples, or perhaps an artifact from the use of different measures employed in these studies. What is apparent, is that for both family members and direct support professionals, moderate to severe levels of stress were more likely to be reported than depression and anxiety. This is an important finding as, to the authors’ knowledge, stress has not been measured in previous research and requires further investigation given the mediating effects among anxiety, depression and stress ( Nima *et al.,* 2013).

A key finding from the present study is the significant difference in stress, depression and anxiety reported between family members and direct support professionals, which in all cases found family members as more likely to be classified in the moderate and severe range of these indicators of wellbeing. It is important to note that this study also found that family members were less likely than direct support professionals to support individuals with co-morbid conditions such as epilepsy, behaviours that challenge and limitations in self-care and communication, which in combination would suggest that direct support professionals are supporting individuals with higher levels of support need. This finding indicates that for caregivers in this study, their wellbeing was not a function of the level of disability of the person they care for, rather other factors contributed. Data from this study identify observed changes in the mood of a family member with IDD as a key contributor to the wellbeing of family caregivers, while staff operations, specifically reorganisation of staff shifts and the introduction of new staff, were key contributors to the wellbeing of direct support staff. 

### Strengths and limitations of the present study

There are a number of key strengths to the present study. It was devised with the support of the membership of IASSIDD’s (International Association for the Scientific Study of Intellectual and Developmental Disabilities) Comparative Policy and Practice Special Interest Research Group who developed the survey in an iterative process, reverse-translated to 15 languages and disseminated among their networks. This level of cooperation by 26 experienced disability researchers attests to the content validity of the survey instrument, translation process, and sharing of the survey among national disability and advocacy organisations. The success of the survey, with 3,754 valid responses, may be considered as proof of concept that global online research can be successfully undertaken.

The funder requirement of adherence to strict data management guidelines was a new departure for all 26 researchers, none of whom had completed a data management plan to the standard required by the funder, nor who had curated a disability dataset for archiving in a data repository. This experience has been valuable, not only given the learnings from a Research Data Manager with considerable expertise in this field, but also given the developments by European Commission ^1^ and US National Institute of Health ^2^ for data management in future research calls where co-investigators may seek research funding.

Finally, the use of a standardised measure of wellbeing, the DASS12, is an important assurance that the key outcome examined in this survey is psychometrically valid. This is particularly important given the differences in wellbeing noted in this study when compared with previous studies.

There are also some limitations to the study which must be considered when interpreting findings. Most notably and commented on by the reviewers of the published study protocol, is the failure to include the voice of individuals with IDD directly. The rationale in the present study not to directly include persons with IDD was that at the time of application for funding, many of the participating countries were in lockdown. Many of the services supporting people with IDD were either closed or greatly reduced which the broader dataset confirms. It was the opinion of the 26 disability researchers supporting this research that disability agencies were completely focused on modifying supports to meet need and on reducing transmission, both of which were impacted by staff absences as many staff were required to self-isolate. To address this limitation, the draft findings were presented to self-advocates with the support of Inclusion International, an advocacy organisation for people with disabilities and their families. Additionally, several co-investigators are now engaged in qualitative studies to capture the direct voice of persons with IDD during the pandemic.

Another limitation of the study is recording only the perceptions of caregivers who had access to online devices, which while common in some countries, will undoubtedly exclude some who are economically challenged. The findings also represent only the views of those who chose to participate, and there may be systematic differences when compared with those who chose not to; notably, the findings are overly representative of high-income countries. Those who did respond have provided their perception of events and observations, which are open to subjectivity and cannot be validated. Family members and direct support professionals for example, who reported a change in mood in the person they support, may in fact be projecting their own suspicions rather than identifying any real change in behaviour. There is evidence of clear disparity between the reporting of research participants’ perceptions and more objective measures of data gathering such as observational data within disability research ( Mansell, 2011). Given the constraints of the present study during a pandemic, objective measures using observational techniques were not possible, and while open to bias, the perceptions of family members and direct support staff are of themselves of interest when examining their wellbeing. Despite these challenges, the dataset from this study is nonetheless an important global perspective from which the experiences of caregivers can be gleaned via their own individual perspective. Although these data are not representative for any participating country, collectively there are findings which, given the sample size, reflect a general trend of the impact of COVID-19 on individuals with IDD and their caregivers. A series of recommendations are presented in Table 21 below.

**Table 21.** Recommendations to ameliorate the findings of a negative impact of COVID-19 for persons with IDD, family members, and those working in the disability field.

Persons with IDD	Family members	Direct support staff and management in disability organisations
Develop timely, accessible, accurate and informative materials on COVID-19 for persons with IDD.	Provide resources for family members on how best to respond if they observe changes in the person they support, for example, in mood and/or behaviours indicative of diminished wellbeing.	Conduct a wide-ranging consultation among disabled persons’ organisations, disability providers, government and other stakeholders regarding the options to avoid the closure of disability services during periods of risk.
Ensure continuity of support services is prioritised during periods of risk. Disability support services must be classified as essential services that cannot be withdrawn.	Address family members’ concerns to contact healthcare providers during periods of risk to address their own or their family members’ health needs.	Confer disability support professionals with essential worker status on a par with other health workers and identify and prepare suitable alternatives if a reduction of existing disability support services is unavoidable during periods of risk.
Ensure engagement with family and friends are always facilitated during periods of risk, if necessary, via online methods.	Develop and implement protocols for family members specifying how to report incidents of exploitation against persons with IDD.	Secure funding from central government for online and other remote options required to ensure continuity of service during periods of risk.
Proactively lobby governments to implement long-term policies to close congregated settings, or introduce such policies if they do not exist, notably given the elevated risk of exposure during pandemics. Where these exist, ensure those living in such settings are prioritised during periods of risk.	Provide guidance to family members to recognise the presentation of COVID- 19 symptoms among persons with IDD taking a ‘higher index of suspicion’ approach.	Develop and implement protocols to guide the re-introduction of disability support services with minimal disruption to persons with IDD.
Develop and implement protocols with healthcare providers to plan for uninterrupted access to healthcare for persons with IDD.	Engage with family members to develop protocols for COVID-19 testing and treatment options for their family member with IDD.	Develop and implement protocols to address any shortage of staff during periods of risk, with reliance on casual staff to breach the gap as an emergency response only.
Triage protocols within acute hospital sector should be reviewed by disability persons’ organisations to avoid discriminatory practices.	Develop and implement protocols for family members accompanying their family member with IDD to hospital to ensure they are identified with the status of support person as opposed to visitor.	Renumerate staff for additional workload during periods of risk.
Engage with individuals with IDD to develop and implement protocols for COVID-19 testing and treatment options.	Disability support services should support family members to ensure a clear plan is available regarding the support of their family member with IDD in the event the caregiver becomes ill.	Develop timely, accessible, accurate and informative materials on COVID-19 for staff working in disability support services.
Ensure that extra costs incurred during periods of risk are covered by central government and local authorities are not taken from disability allowances and/or personal budgets of individuals or family members of persons with IDD.	Develop timely, accessible, accurate and informative materials on COVID-19 for family members.	Address the reluctance by some direct support staff to report incidents of exploitation against persons with IDD.
Develop and implement protocols for the inclusion of disability variables, as per the guidance of the UN Washington Group, in routine national and international health interview and health examination surveys.	Liaise with unions and employers regarding the duties of employees who are caregivers and may require paid time- off from work.	Develop and implement protocols to ensure timely access to PPE, isolation and testing facilities.
	Liaise with unions and employers regarding the possibility of flexible working hours to accommodate employees who are caregivers.	Facilitate access to psychological supports for staff working in disability support services.
	Facilitate access to psychological supports for family caregivers.	Support research to explore factors contributing to high levels of stress, depression and anxiety among disability staff which can inform interventions to ameliorate distress.
	Support research to explore factors contributing to high levels of stress, depression and anxiety among family members which can inform interventions to ameliorate this distress.	

## Conclusion

The findings of this study reveal disrupted access for persons with IDD to support services and healthcare, and engagement in behaviours indicative of mental health distress. These are important findings within the context of the UN Convention on the Rights of Persons with Disabilities which requires signatories to take all necessary measures to ensure the protection and safety of persons with disabilities in situations of risk.

In contrast to the widespread acknowledgement of the burden of COVID-19 on older persons there has been insufficient recognition of the impact of the pandemic on persons with disabilities, especially those who are resident in congregated settings ( Comas-Herrera *et al.,* 2020). A policy response is urgently required in many international jurisdictions to redress the lack of action to date. This policy should be cognizant of the fact that the support needs model of disability advocated by the American Association for Intellectual and Developmental Disability argues that supports are essential to ensure quality of life outcomes for persons with IDD ( American Association for Intellectual and Developmental Disability, 2021). To infringe on these supports, to close and reduce services, is to knowingly fail to recognise that such services are not an inconvenience when lost, rather they are essential and necessary, and their removal is likely to cause long-term negative impact on individuals’ wellbeing. While not included in this survey, the direct voice of people with IDD is urgently needed to provide a true picture of the impact of the withdrawal of these services.

Also revealed in this study are high levels of stress, depression, and anxiety of caregivers, many of whom experienced economic hardship and increased workload during a time where they observed the negative impact of the pandemic on those they support as a direct consequence of lockdown restrictions. It is worth noting that over 80% of participants in the present study were women, a common pattern observed in disability research. Their experiences add to a growing body of research highlighting the disproportionate impact of the pandemic on women ( Fuith & Trombley, 2020). Policies are urgently required to address the evidenced burden of care placed on caregivers, many women, who work tirelessly in the pursuit of positive quality outcomes for the persons they support. 

Consideration is also required to the address the inability of many disability researchers to undertake secondary data analysis of large population-based survey to determine the impact of the pandemic on persons with disability. Population based data would have enabled an examination of the long-term effects of the pandemic on this population over different waves of COVID-19 since the present data were collected. For almost 30 years, the United Nations’ Washington Group on Disability has worked on the development of disability items that can be included in such datasets to enable the extraction of disability data for analysis. Despite these developments, many omnibus health interviews and health examination surveys fail to include persons with disabilities who consequently remain invisible when these datasets are used to guide public health policy and practice ( Linehan *et al.,* 2009). At a time of pandemic, when population-based data are urgently required, the failure to include people with disabilities in population-based datasets is discriminatory and requires immediate attention.

## Data availability

### Underlying data

Open Science Framework: COVID-19 IDD: a global survey exploring family members’ and paid staffs’ perceptions of the impact of COVID-19 on individuals with intellectual and developmental disabilities and their caregivers. https://doi.org/10.17605/OSF.IO/GK2VF. ( Linehan *et al.,* 2022).

This project contains the following underlying data:

- 4. COVID_19_IDD_DATA FILE. SAV (This is an SPSS file containing the anonymised data from 3,754 family members and paid staff who supported individuals with intellectual and developmental disabilities in 12 countries worldwide).

Data are available under the terms of .0

### Extended data

Open Science Framework: COVID-19 IDD: a global survey exploring family members’ and paid staffs’ perceptions of the impact of COVID-19 on individuals with intellectual and developmental disabilities and their caregivers. https://doi.org/10.17605/OSF.IO/GK2VF. ( Linehan *et al.,* 2022).

This project contains the following extended data:

- 1. COVID_19_IDD Naming convention of variables. (This MS Word file provides information on the convention used to name variables in the SPSS datafile).
- 2. COVID_19_IDD Data dictionary in order of survey. (This EXCEL file identifies the variable and value labels for variables in the SPSS datafile).
- 3. COVID_19_IDD_Qualtrics Version of Survey. (This MS Word file presents all questions on the survey in the exact format they were presented on Qualtrics. Variables included in the survey but excluded from the SPSS datafile during the anonymisation process are retained in this file.
- 5. COVID_19_IDD_listing of deleted or recoded variables. (This MS Word file lists all variables included in the survey that we either deleted or recoded from the SPSS datafile during the anonymisation process.

Data are available under the terms of the Creative Commons Attribution 4.0 International license (CC-BY 4.0).

A three-month and final data management plan (DMP), prepared by Gail Birkbeck, Research Data Manager is posted to DMPonline ( https://www.dmponline.dcc.ac.uk)

### Reporting guidelines

Open Science Framework: CHERRIES checklist for ‘COVID-19 and IDD: a global survey exploring family members’ and paid staffs’ perceptions of the impact of COVID-19 on individuals with intellectual and developmental disabilities and their caregivers’. https://doi.org/10.17605/OSF.IO/GK2VF.

Data are available under the terms of the Creative Commons Attribution 4.0 International license (CC-BY 4.0).

## Author contributions

Christine Linehan, Conceptualisation, Formal Analysis, Funding Acquisition, Investigation, Methodology, Project Administration, Supervision, Visualisation, Writing – Original Draft Presentation, Writing – Review and Editing

Gail Birkbeck, Conceptualisation, Data Curation, Formal Analysis, Investigation, Methodology, Project Administration, Supervision, Visualisation, Writing - Review and Editing

Tal Araten-Bergman, Conceptualisation, Investigation, Methodology, Writing – Review and Editing

Jennifer Baumbusch, Conceptualisation, Investigation, Methodology, Writing – Review and Editing

Julie Beadle-Brown, Conceptualisation, Investigation, Methodology, Writing – Review and Editing

Christine Bigby, Conceptualisation, Investigation, Methodology, Writing – Review and Editing

Visualisation, Writing – Original Draft Presentation, Writing – Review and Editing

Valerie Bradley, Conceptualisation, Investigation, Methodology, Writing – Review and Editing

Michael Brown, Conceptualisation, Investigation, Methodology, Writing – Review and Editing

Femmianne Bredewold, Conceptualisation, Investigation, Methodology, Writing – Review and Editing

Masauso Chirwa, Conceptualisation, Investigation, Methodology, Writing – Review and Editing

Jialiang Cui, Conceptualisation, Investigation, Methodology, Writing – Review and Editing

Marty Godoy Gimenez, Conceptualisation, Investigation, Methodology, Writing – Review and Editing

Tiziano Gomiero, Conceptualisation, Investigation, Methodology, Writing – Review and Editing

Sarka Kanova, Conceptualisation, Investigation, Methodology, Writing – Review and Editing

Thilo Kroll, Conceptualisation, Investigation, Methodology, Writing – Original Draft Presentation, Writing – Review and Editing

Henan Li, Investigation, Writing – Review and Editing

Mac MacLachlan, Conceptualisation, Investigation, Methodology, Writing – Review and Editing

Jayanthi Narayan, Conceptualisation, Investigation, Methodology, Writing – Review and Editing

Finiki Nearchou, Conceptualisation, Investigation, Methodology, Writing – Review and Editing

Adam Nolan, Conceptualisation, Investigation, Methodology, Writing – Review and Editing

Mary-Ann O’Donovan, Conceptualisation, Investigation, Methodology, Writing – Review and Editing

Flavia H Santos, Conceptualisation, Investigation, Methodology, Writing – Review and Editing

Jan Siska, Conceptualisation, Investigation, Methodology, Writing – Review and Editing

Tim Stainton, Conceptualisation, Investigation, Methodology, Writing – Review and Editing

Magnus Tideman, Conceptualisation, Investigation, Methodology, Writing – Review and Editing

Jan Tossebro, Conceptualisation, Investigation, Methodology, Writing – Review and Editing
