# Supplementary material for: COVID-19 IDD: Findings from a global survey exploring family members’ and paid staff’s perceptions of the impact of COVID-19 on individuals with intellectual and developmental disabilities (IDD) and their caregivers
Source: HRB Open Res. 2022 Apr 4;5:27. [Version 1] doi: 10.12688/hrbopenres.13497.1 (PMC9111363; doi:10.12688/hrbopenres.13497.1)
Supplement: Peer review history

# Peer review: Reviewer response for version 1

**DOI:** 10.21956/hrbopenres.14721.r31882

**Published:** 2022-05-16

**Authors:** Henrietta Trip

**Affiliations:** 1 Centre for Postgraduate Nursing Studies, University of Otago, Christchurch, New Zealand

Congratulations and thank you for bringing together this international collaboration to identify the unique global experiences of family carers and direct support professionals in supporting and responding to people with intellectual and developmental disabilities (IDD) in the early stages of the COVID-19 pandemic.

 This research encompassing Europe, North America, and the Asia-Pacific regions clearly reiterates the continued lack of equity afforded to and experienced by people with IDD and those in their network of support in regard to healthcare access; inclusion in public health planning, advice, and implementation; and health and disability service delivery that actively incorporates reasonable adjustments. These identified global gaps equate to an infringement of human rights and evidence the lack of address required by all signatory countries under the United Nations Convention on the Rights of Persons with Disabilities – particularly under Article 25 Health.

 A transparent and replicable E-survey process has been presented and is evidenced by robust statistical analysis, interpretation, and application of the results. These findings have relevance to individuals with IDD, family carers, service providers and their staff, as well as healthcare systems globally, given the ramifications for the health and wellbeing of people with IDD.

 Recommendations that may inform the international readership beyond the IDD community:

- Page 6, para 1: Delete repeated sentence at the end – ‘and questions relating to… treatment.’
- In the abstract and throughout the article, reference is made to families, paid staff, direct support professionals, carers, and caregivers. Whilst the latter two terms represent all parties, readers from other sectors may be unclear about the difference between paid staff and direct support professionals (DSP); hence, it is suggested that these be defined and one consistently used in the article.
- Keywords – Include ‘direct support professionals’ and consider whether both ‘carers’ and ‘caregivers’ are needed.
- Reference is made to the languages of translation – check ‘Sweden’.
- There is an opportunity to inform interpretation for the wider readership: Consider using the term ‘presentations or behaviours that challenge’ rather than ‘challenging behaviour’. The latter often implies that which is intrinsic to the person, that the locus of control sits with them. The former phrase reflects that extrinsic factors play a key role and impact upon how a person may understand, respond, and present under varying circumstances.

The discussion and conclusion identify the links between what was known about the health of people with IDD and what the COVID-19 pandemic has highlighted. Furthermore, it clearly both petitions and demands socio-political action, not only from jurisdictions but the international community to redress the continued and evolving disparities experienced by people with IDD and those in their networks of support.

Is the work clearly and accurately presented and does it cite the current literature?

Yes

If applicable, is the statistical analysis and its interpretation appropriate?

Yes

Are all the source data underlying the results available to ensure full reproducibility?

Yes

Is the study design appropriate and is the work technically sound?

Yes

Are the conclusions drawn adequately supported by the results?

Yes

Are sufficient details of methods and analysis provided to allow replication by others?

Yes

Reviewer Expertise:

In the field of intellectual and developmental disability: social models and accessibility, health promotion, long-term conditions and self-management approaches, workforce development, disability rights and autonomy.

I confirm that I have read this submission and believe that I have an appropriate level of expertise to confirm that it is of an acceptable scientific standard.

# Peer review: Reviewer response for version 1

**DOI:** 10.21956/hrbopenres.14721.r31797

**Published:** 2022-04-22

**Authors:** Germain Weber

**Affiliations:** 1 Department of Clinical and Health Psychology, Faculty of Psychology, University of Vienna, Vienna, Austria

**Strengths:**

- Global sample approach, including multiple countries from Europe, Asia, Oceania, and Northern America supporting general trends, beyond publications with data based on a single country or jurisdiction.
- The application of guidelines for E-surveys (CHERRIES) is widely accepted within the social science community.
- Perceptions of caregivers (family members and paid staff) on COVID-19 effects, such as disparities, specific risks, and well-being of people with intellectual and developmental disabilities as well as COVID-19 effects on caregiver’s well-being.
- Robust statistical analysis, including logistic linear regression analysis.
- Findings reveal areas to be urgently addressed in socio-political considerations and actions on a national or regional level.

**Suggestions for improvement:**

- Add "human rights" to the keyword list.
- Address more strongly the fact that the findings refer to experiences during the first 6-7 months of the pandemic in the conclusion, issues of psychological adaptation to a new challenging situation.
- Check for Swedish, in the section addressing the languages of the questionnaire.
- Statistical analysis: Better to make use of the number of persons with disabilities living in the accommodation where the responding carer is offering support instead of making use of the overall figure of persons with disabilities supported at different locations by a service provider.
- Reflect on the fact that COVID-19 may have contributed to being more sensitive in reporting incidents, i.e. incidents reported might have existed prior to the pandemic.
- When addressing data analysis (8), the authors report introducing three new variables to control of severity of COVID-19 and restrictions imposed in jurisdictions. This level of analysis is not addressed in the results, thus, it might be deleted in section (8).     
- As the sample is not representative, the conclusion referring to 80% of women having engaged in this survey should be presented in a modified way!

Overall, kudos to the consortium and its achievement!

Is the work clearly and accurately presented and does it cite the current literature?

Yes

If applicable, is the statistical analysis and its interpretation appropriate?

Yes

Are all the source data underlying the results available to ensure full reproducibility?

Yes

Is the study design appropriate and is the work technically sound?

Yes

Are the conclusions drawn adequately supported by the results?

Partly

Are sufficient details of methods and analysis provided to allow replication by others?

Yes

Reviewer Expertise:

Psychology of intellectual disability, self-determination theory, human rights

I confirm that I have read this submission and believe that I have an appropriate level of expertise to confirm that it is of an acceptable scientific standard.
